# Ferrocene and Transition Metal Bis(Dicarbollides) as Platform for Design of Rotatory Molecular Switches

**DOI:** 10.3390/molecules22122201

**Published:** 2017-12-11

**Authors:** Igor B. Sivaev

**Affiliations:** A. N. Nesmeyanov Institute of Organoelement Compounds, Russian Academy of Sciences, 28 Vavilov Str., Moscow 119991, Russia; sivaev@ineos.ac.ru; Tel.: +7-916-590-2025

**Keywords:** molecular switches, ferrocene, transition metal bis(dicarbollides)

## Abstract

Design of rotatory molecular switches based on extremely stable sandwich organometallic complexes ferrocene and bis(dicarbollide) complexes of transition metals is reviewed. The “on”–“off” switching in these systems can be controlled by various external stimuli such as change of the solution pH, interactions with coordinating species or redox reactions involving the central atom or substituents in the ligands.

## 1. Introduction

The design of molecular machines is a very important area of research, which has been growing extraordinarily fast during the past few decades and was recognized by the award of the Nobel Prize in Chemistry in 2016 to Sauvage, Stoddart, and Feringa “for the design and synthesis of molecular machines” [1,2,3,4,5]. On the basis of the type of motion, molecular machines can be divided into two main types, that is, molecular motors and molecular switches. Molecular motors are molecular machines that are capable of unidirectional rotation motion by 360° powered by external energy input, whereas molecular switches are molecules or supramolecular complexes that can exist in two or more stable forms that differ in the mutual orientation of their components and can be converted readily from one state to another by various external stimuli such as heat, light, and chemical reagents. Molecular switches are the main structural element of any molecular electronics devices, particularly molecular logic gates, where the combination of two or more molecular switches allows molecule to behavior like that of electronic logic gates, suggesting a basis for future nanosize computing devices [6]. Therefore, the design and the study of molecules and supramolecular systems capable of performing mechanical movement is an important and urgent problem of modern chemistry. Currently, the most studied are photochromic molecular switches based on azobenzenes, stilbenes, dithienylethenes, spiropyrans and spirooxazines, switches based on the “host–guest” interactions, and switches based on mechanically-interlocked molecular architectures, rotoxanes and catenanes, where the bistable states differ in the position of the macrocycle [7]. Despite the noteworthy progress in the synthesis and study of such molecules, there are some problems caused by the relatively low stability of many organic materials to atmospheric oxygen and moisture, and this has stimulated the search for new types of compounds as structure-forming components in the design of effective molecular switches. Therefore, there is growing interest in molecular switches based on transition-metal complexes [8]. However, as in the case of organic molecular machines, devices based on transition metals complexes often have a low stability. Therefore, the choice of a suitable stable organometallic unit is of significant importance for the development of molecular electronics devices.

In this contribution we review the main achievements in the design of rotarory molecular switches on the base of extremely stable sandwich organometallic complexes—ferrocene and transition metal bis(dicarbollides).

## 2. Ferrocene Based Molecular Switches

The archetypal organometallic compound ferrocene [(η^5^-C_5_H_5_)_2_Fe] is of historical importance since its discovery and structural characterization in the early 1950s sparked extensive research into the chemistry of metal sandwich compounds [9]. The iron(II) ion in ferrocene is sandwiched between the two cyclopentadenyl ligands (*Cp*) and acts as “atomic ball-bearing” enabling nearly free rotation of the ligands (the rotation barrier is approx. 1.1 kcal/mol with the eclipsed conformation preferred [10]). The short distance between the planes of the cyclopentadenyl ligands (3.3 Å) results in close proximity of substituents in 1,1′-disubstituted ferrocenes, therefore the substituents that are able to engage in various intramolecular interactions, such as hydrogen bonding or π–π interactions, favor the formation of the *cisoid* conformation. Since such interactions usually are rather weak, some external actions, e.g., the change of the solution pH, interaction with external metals or redox reactions in substituents, could result in the controllable switching between the *cisoid* and *transoid* conformations that can be used for the design of the “on”–“off” switches. This field was recently briefly covered by Scottwell and Crowley [11] and in this review we will try to give some deeper and more comprehensive perspectivers on this subject.

### 2.1. Protonation-Driven Molecular Switches

**Scheme 1:**
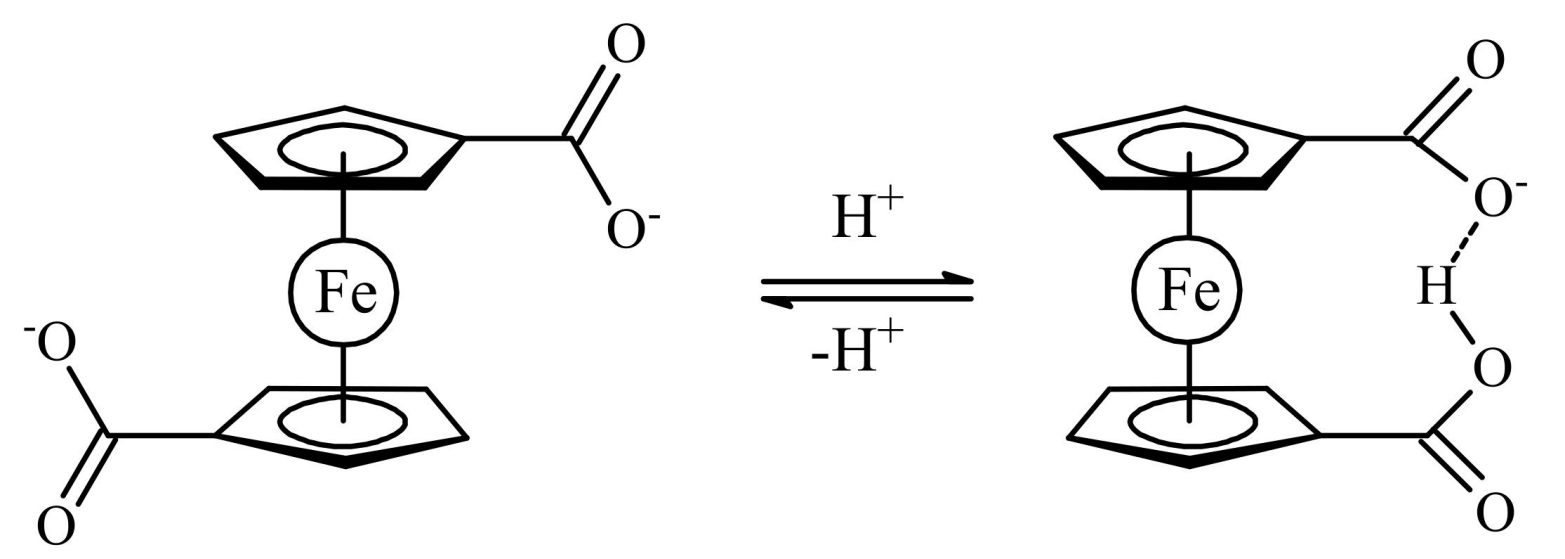
Principle of operation of molecular switch based on 1,1′-ferrocene dicarboxylic acid.

One of the simplest examples of such ferrocene-based switches which was proposed by Wang et al. is 1,1′-ferrocene dicarboxylic acid [12]. In the fully deprotonated form, the two negatively charged carboxylates electrostatically repel one another causing rotation of *Cp* ligands to give the *transoid* conformation. Protonation of one carboxylate group results in stabilization of the *cisoid*-like conformation due to hydrogen bonding between the two carboxylate groups with the energy minimum of 60 kJ/mol (rotation angle is 68°). The deprotonation causes the system to switch back to its initial *transoid* state (Scheme 1). It should be noted that despite low energy minimum of the *transoid* conformation for the fully deprotonated form (145 kJ/mol), this minimum is rather wide with difference in energy of the *transoid* and *gauche* rotamers being only 30 kJ/mol [12]. However, all these data concern gas-phase structures, whereas in solution and in the solid state the preference of different rotomers depends on interactions of the substituents with solvent molecules or neighbor ions/molecules in the crystal lattice. In the solid state, the *cisoid* conformation of 1,1′-ferrocene dicarboxylic acid is stabilized by the formation of hydrogen-bonded dimers rather than intramolecular hydrogen bonding between the substituents in different *Cp* ligands [13,14]. The conformation of the ferrocene fragment both in mono- and in dicarboxylate salts depends strongly on the counter ion and its ability to form supramolecular structures [15], whereas the conformation of ferrocene in various complexes with 1,1′-ferrocene dicarboxylate ligand is determined by a choice of the transition metal cation and additional ligands [16,17,18,19,20,21,22,23,24].

**Scheme 2:**
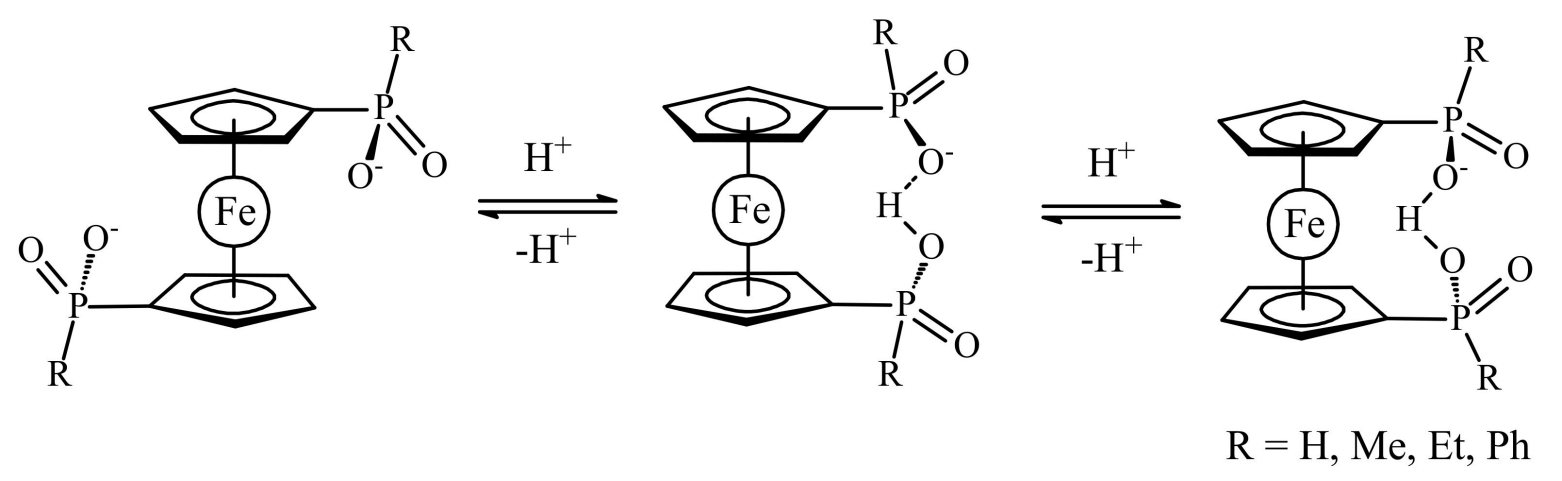
Principle of operation of molecular switch based on 1,1′-ferrocene diphosphinic acids.

**Figure 1:**
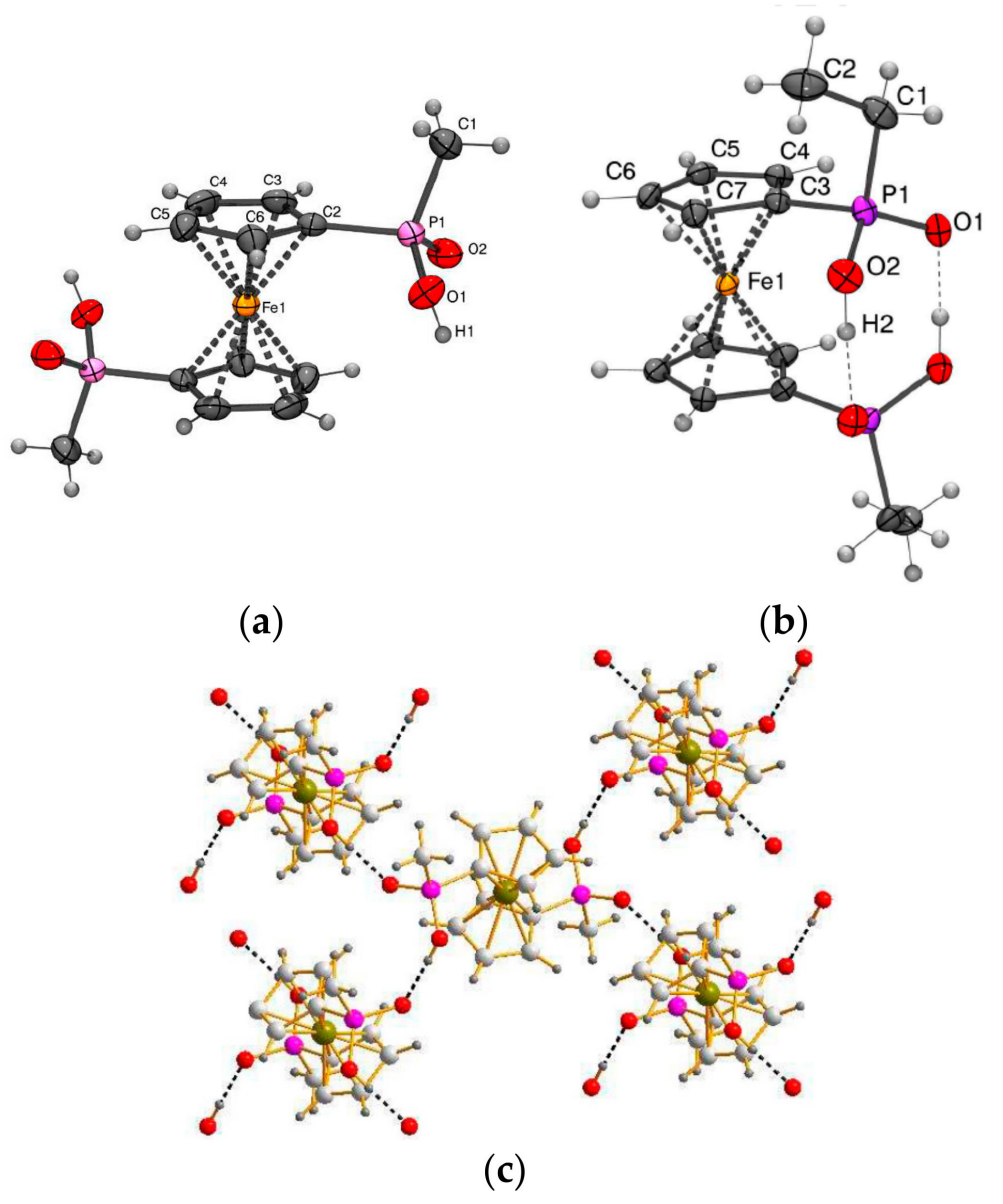
X-ray crystal structures of ferrocene-1,1′-diyl bis(methylphosphinic) (**a**) and ferrocene-1,1′-diyl bis(ethylphosphinic) (**b**) acids and fragment of crystal packing of ferrocene-1,1′-diyl bis(methylphosphinic) acid (**c**). Reprinted with permission from [26]. Copyright (2014) Elsevier (Amsterdam, The Netherlands).

**Figure 2:**
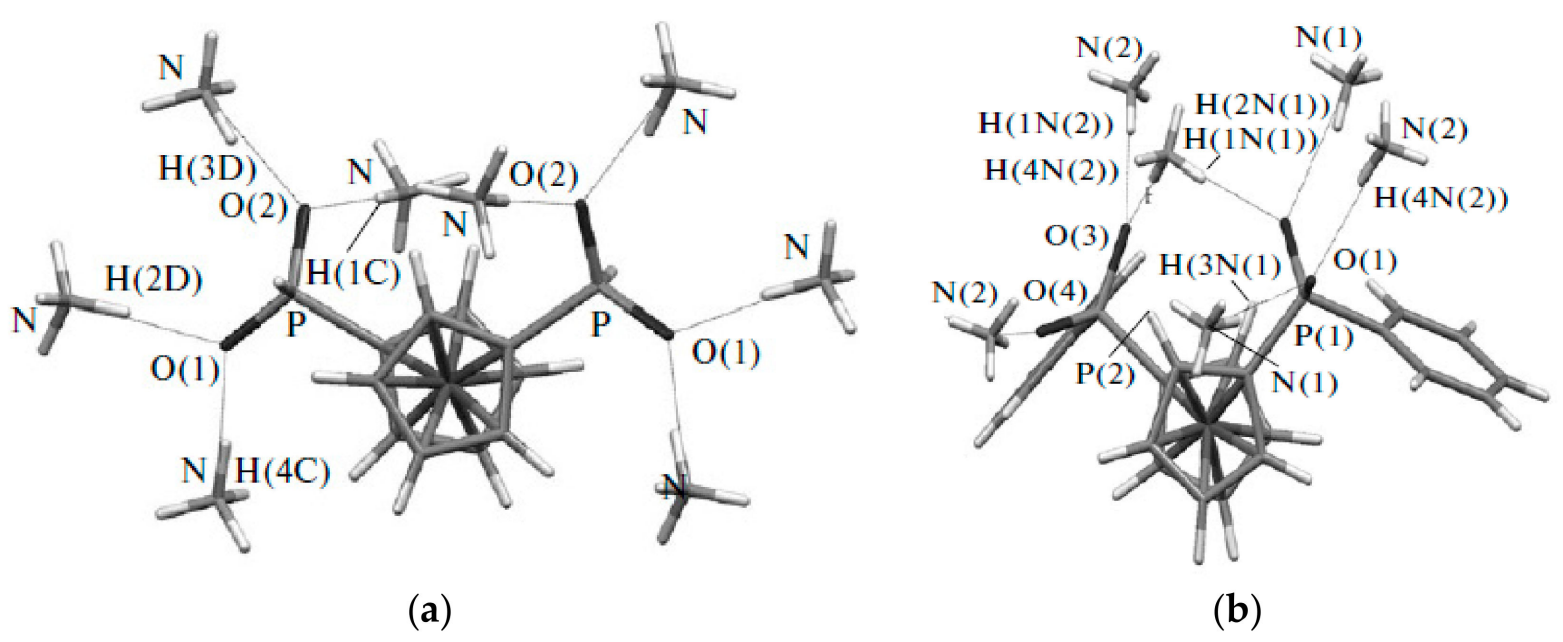
X-ray crystal structures of diammonium ferrocene-1,1′-diyl bis(phosphinate) (**a**) and diammonium ferrocene-1,1′-diyl bis(phenylphosphinate) (**b**). Reprinted with permission from [27]. Copyright (2014) Springer Science (Berlin, Germany).

Recently 1,1′-ferrocene diphosphinic acids were proposed as similar proton-driven switchers [25]. In the neutral, fully protonated form, the eclipsed *cis*-conformation (rotation angle is 1.5°) is stabilized by two intramolecular hydrogen bonds between the phosphinic groups. The removal of one proton results in breaking one hydrogen bond with transformation to the *cisoid* conformation (rotation angle is ~45°). In the fully deprotonated form, the two negatively charged phosphinates electrostatically repel one another to give the *transoid* conformation (Scheme 2). Similar to 1,1′-ferrocene dicarboxylic acid, in the solid state the preference of different rotomers is determined by the competition of intermolecular and intramolecular hydrogen bonding. For example, the *transoid* conformation was found in the solid state for ferrocene-1,1′-diyl bis(methylphosphinic) acid (η^5^-C_5_H_4_P(O)(Me)OH)_2_Fe, whereas in the cases of ferrocene-1,1′-diyl bis(ethylphosphinic) (η^5^-C_5_H_4_P(O)(Et)OH)_2_Fe and ferrocene-1,1′-diyl bis(phenylphosphinic) (η^5^-C_5_H_4_P(O)(Ph)OH)_2_Fe acids the expected *cis*-conformation is realized (Figure 1) [26]. The conformation in the diammonium salts of ferrocene-1,1′-diyl bis(phosphinic) and ferrocene-1,1′-diyl bis(phenylphosphinic) acids is determined by the interactions of the substituents with ammonium cations in the crystal lattice, where the respective rotation angles of ~120° and ~90° were found (Figure 2) [27].

**Scheme 3:**
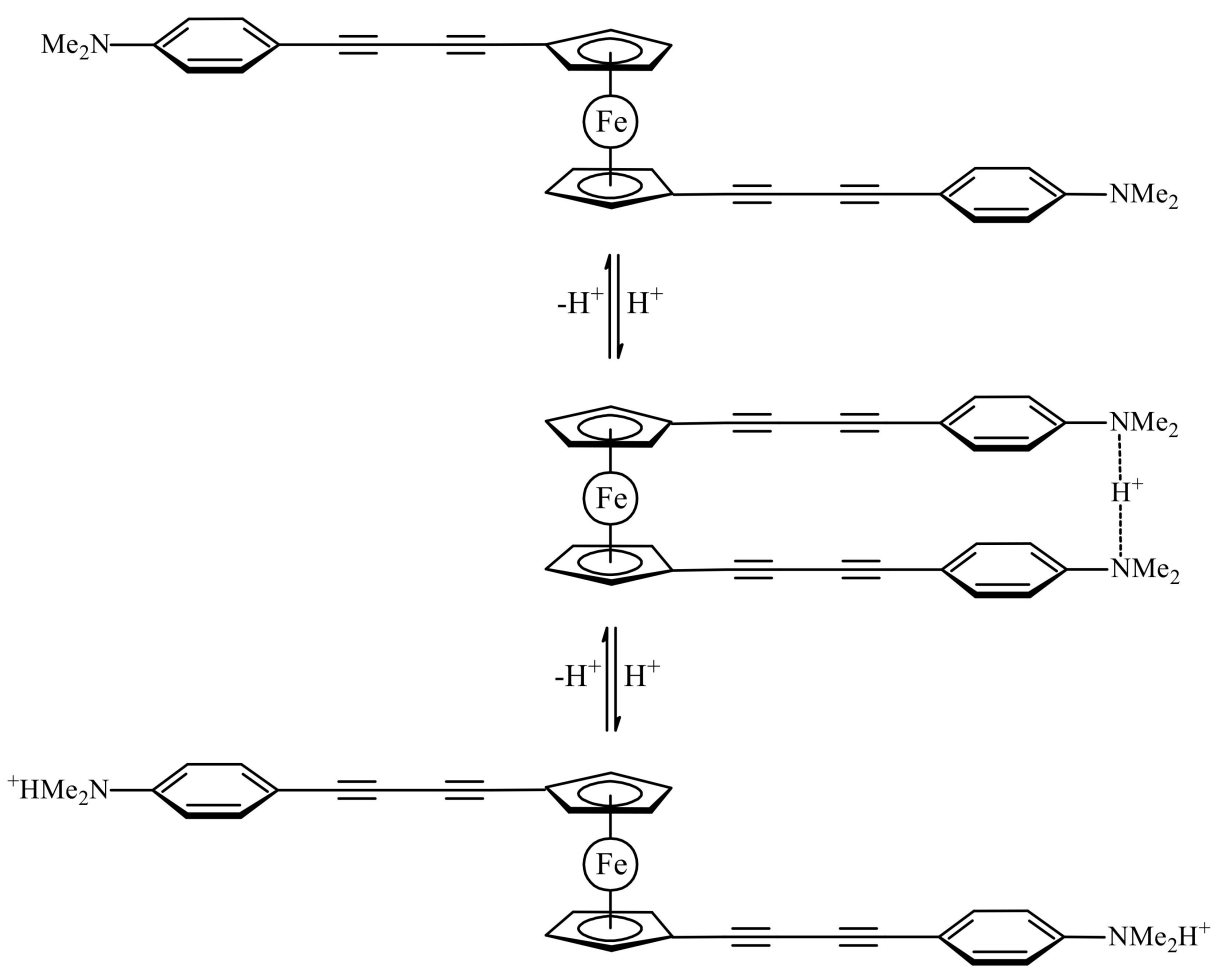
Operation principle of molecular switch based on protonation–deprotonation of 1,1′-bis(4-(*N*,*N*-dimethylaminophenyl)butadiynyl)ferrocene.

1,1′-Bis(4-(*N*,*N*-dimethylaminophenyl)butadiynyl)ferrocene gives another example of hydrogen bond assisted stabilization of the *cis*-conformation of ferrocene. The protonation of one nitrogen leads to the formation of the N…H…N bridge hydrogen bond between two dimethylaniline fragments in the *cis*-conformation where two *N*,*N*-dimethylaniline groups act as a proton sponge, whereas the protonation of the second nitrogen results in electrostatic repulsion of two positively charged groups to give the *transoid* conformation (Scheme 3). The formation of the N…H…N hydrogen bond-stabilized *cis*-conformation was supported by the ^1^H-NMR and UV spectral data, but exact conformations of the free basic and deprotonated forms are unknown [28].

**Figure 3:**
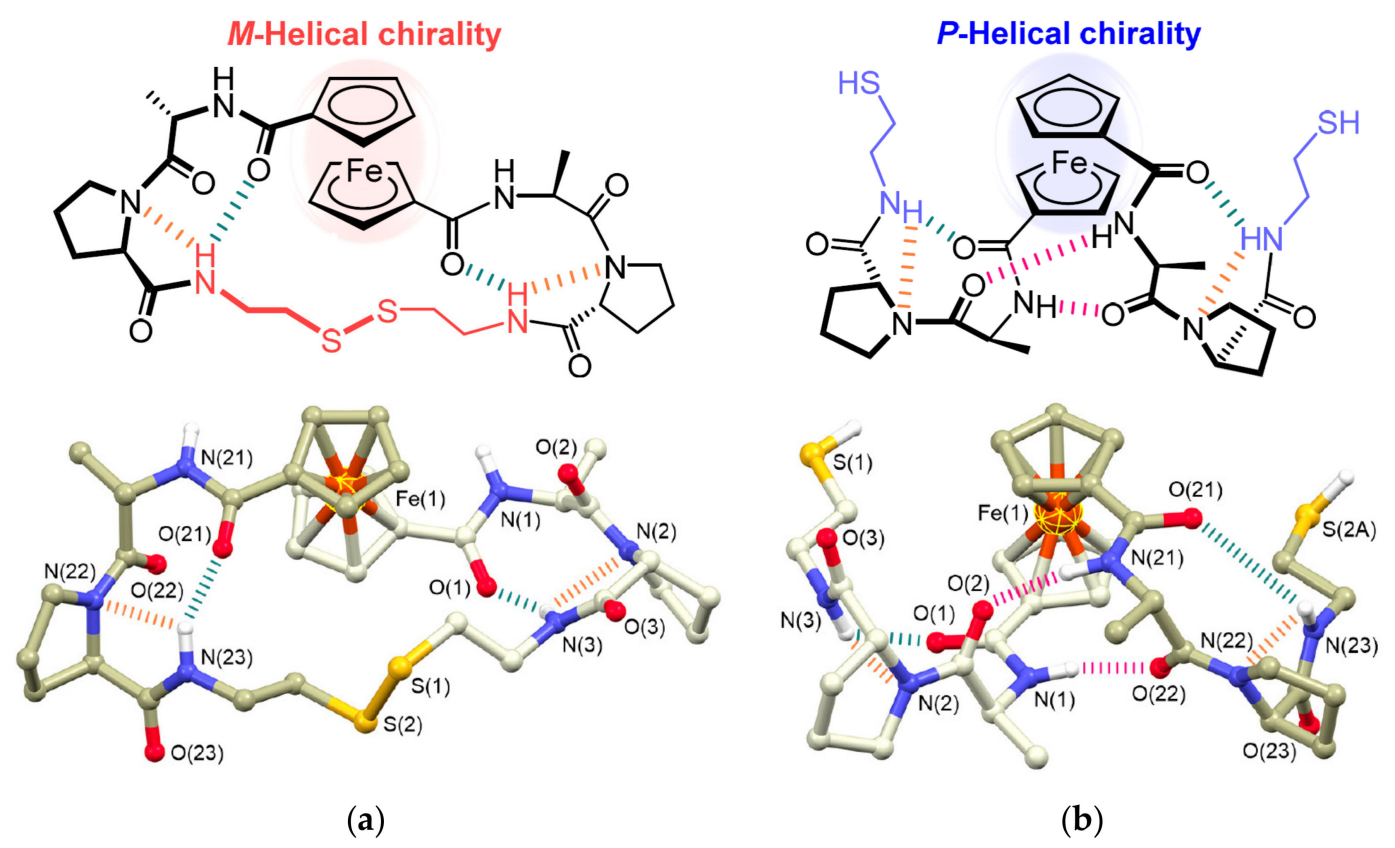
Design (above) and X-ray crystal structures (below) of Fc(-l-Ala-d-Pro-)_2_Cystamine (**a**) and its opened form Fc(-l-Ala-d-Pro-NHCH_2_CH_2_SH)_2_ (**b**). Reprinted with permission from [31]. Copyright (2017) John Wiley and Sons (Hoboken, NJ, USA).

The intramolecular hydrogen bonding between cyclopentadienyl ligands bearing optically active substituents such as dipeptides is known to able to stabilize ferrocene rotamers with different chirality [29,30]. This property can be used for the design of chiroptical molecular switches. The fascinating example is cyclic ferrocene–dipeptide conjugate comprising the l-Ala-d-Pro-cystamine-d-Pro-l-Ala dipeptide sequence. In the solid state this conjugate adopts *M*-helical chirality with *M*-1,4′ helical conformation of the ferrocenyl moiety that is stabilized by the formation of intramolecular hydrogen bonds between the NH group of cystamine and the nitrogen of Pro and the CO group of the ferrocene moiety of each dipeptide moiety. The reductive cleavage of the disulfide bond produces the ring-opened ferrocene derivative bearing two l-Ala-d-Pro-NHCH_2_CH_2_SH dipeptide chains. In the solid state the intramolecular hydrogen bonds are formed between the NH of Ala and the CO of Ala (another chain) of each dipeptide chain to induce *P*-helical chirality with *P*-1,2′ helical conformation of the ferrocenyl moiety (Figure 3). The chirality-organized structures as observed in the crystal structure was found to be preserved even in solution. It should be noted that the construction of such chiroptical molecular switches requires a very careful design of hydrogen bonding. For example, a similar conjugate with the l-Ala-l-Pro-cystamine-l-Pro-l-Ala dipeptide sequence demonstrates P-helical chirality both in cyclic and open form [31].

**Figure 4:**
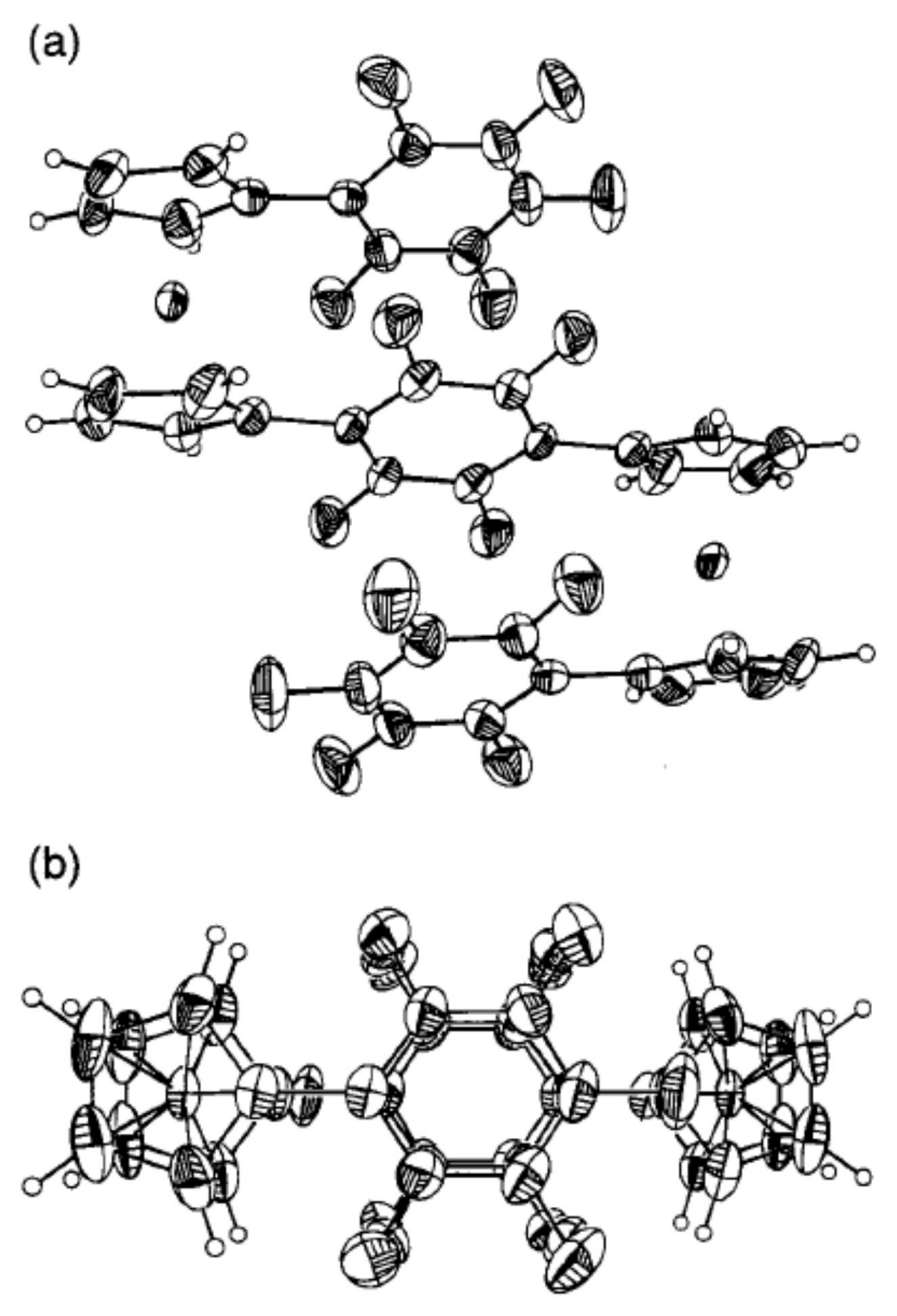
X-ray crystal structure of (η^5^-C_5_H_4_C_6_F_5_)Fe(μ-η^5^-C_5_H_4_-1,4-C_6_F_4_-η^5^-C_5_H_4_)Fe(η^5^-C_5_H_4_C_6_F_5_) (side view (**a**) and view from above (**b**). Reprinted with permission from [32]. Copyright (2010) American Chemical Society (Washington, DC, USA).

Another approach to stabilization of *cis*-conformation in ferrocene derivatives is based on π–π interactions between aryl substituents in 1,1′-diaryl ferrocenes. One of the first and perhaps the most spectacular example of such stabilization represents bis(pentafluorophenyl)-substituted 1,4-tetrafluorophenylene-linked diferrocene (η^5^-C_5_H_4_C_6_F_5_)Fe(μ-η^5^-C_5_H_4_-1,4-C_6_F_4_-η^5^-C_5_H_4_)Fe-(η^5^-C_5_H_4_C_6_F_5_) with two ferrocene fragments in the “closed” *cis*-conformation due to the π–π interactions between three stacked phenyl rings (Figure 4) [32].

**Figure 5:**
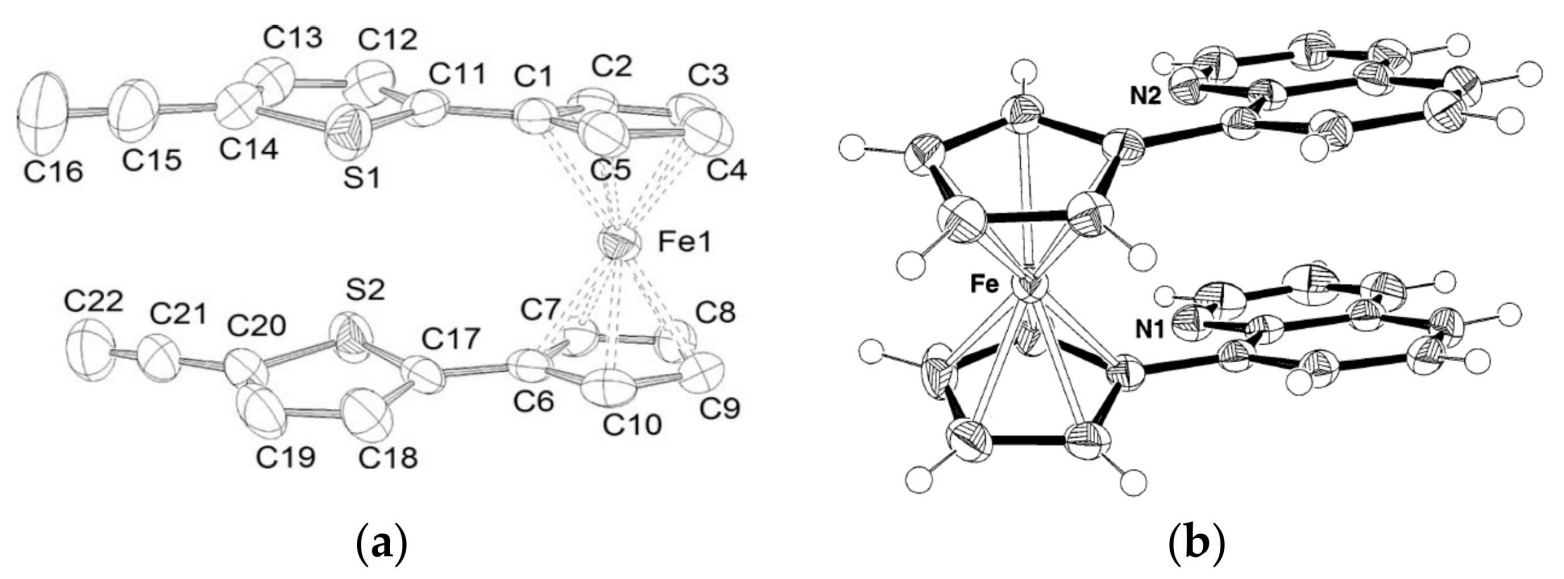
X-ray crystal structures of (η^5^-C_5_H_4_-2-SC_4_H_2_-4-C≡CH)_2_Fe (**a**) and (η^5^-C_5_H_4_-8-NC_7_H_6_)_2_Fe (**b**). Reprinted with permission from [33,34]. Copyright John Wiley and Sons (2010) and Elsevier Science (2001), respectively.

The same type of stabilization was found in 1,1′-bis(heteroaryl) derivatives of ferrocene [33,34,35,36]. Noteworthy, that 1,1′-bis(5-ethynyl-2-thienyl)ferrocene crystallizes in the π-stacked *rac*-conformation (Figure 5a) [33], whereas 1,1′-bis(8-quinolyl)ferrocene adopts the π-stacked *meso*-conformation (Figure 5b) [34].

**Scheme 4:**
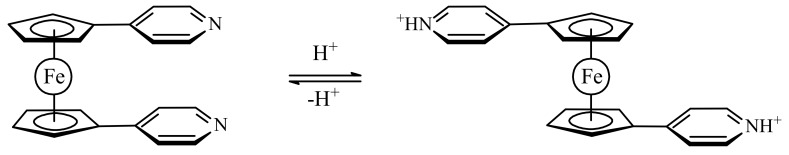
Proposed operation principle of molecular switch based on protonation–deprotonation of π-stacked basic heteroaromatic substituents.

Bosnich et al. supposed that protonation of basic heteroaromatic substituents attached to ferrocene should produce their mutual and generate controlled rotational motion resulting in the formation of a two-state switch (Scheme 4) [35].

**Figure 6:**
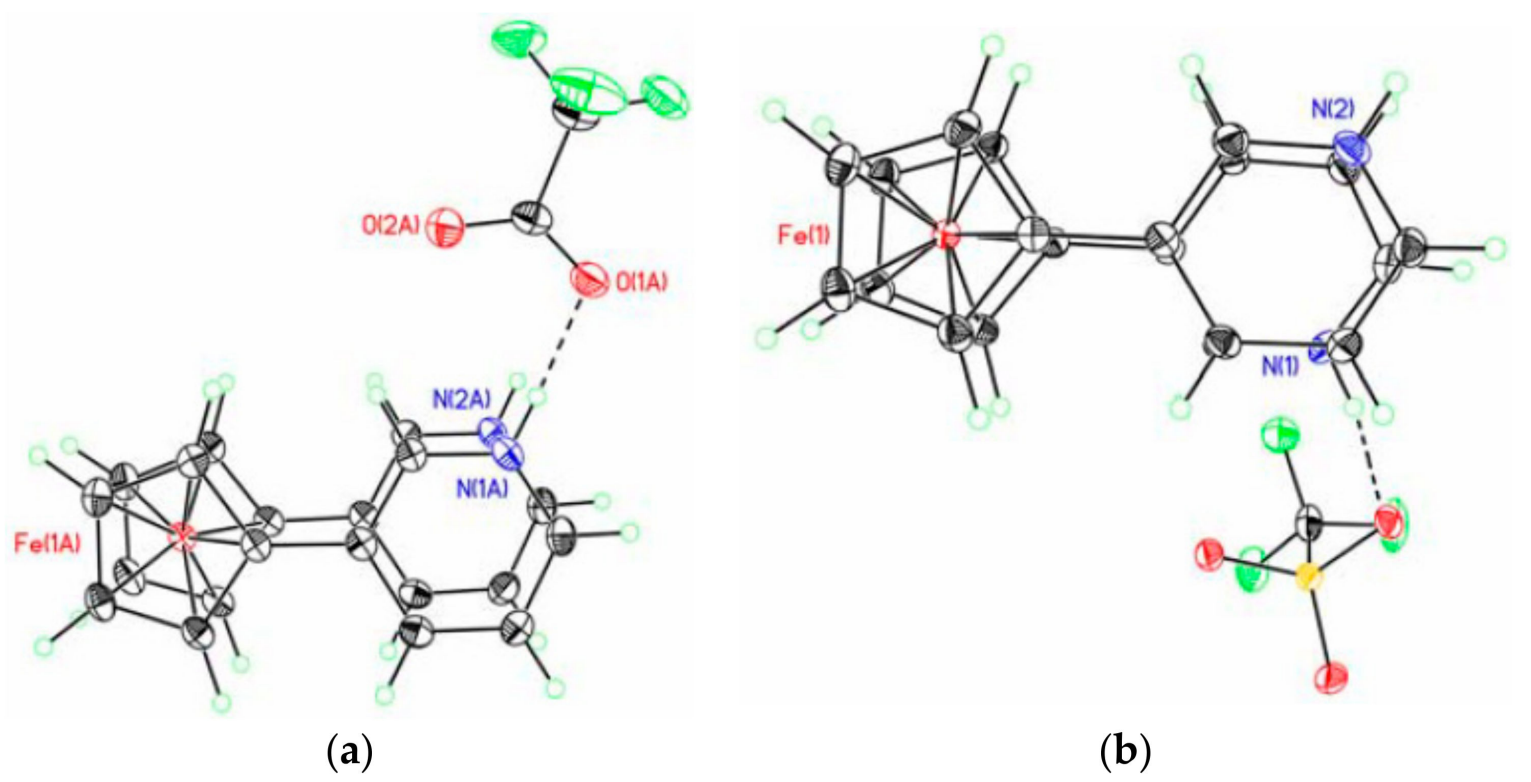
X-ray crystal structures of [(η^5^-3-HNC_5_H_4_-C_5_H_4_)_2_Fe](TFA)_2_ (**a**) and [(η^5^-3-HNC_5_H_4_-C_5_H_4_)_2_Fe](OTf)_2_ (**b**). Reprinted with permission from [35]. Copyright (2010) John Wiley and Sons.

The two pyridine substituents in 1,1′-bis(3-pyridyl)ferrocene are π-stacked. It was expected that their protonation should cause them to electrostatically repel one another and rotate away from each other. However, when trifluoroacetic and triflic acids were used to protonate the pyridines and generate positive charge on the substutients, in the solid state the both anions was found to form strong hydrogen bond to the pyridine N-H, thus quenching the charges and resulting in no electrostatic repulsion and (Figure 6) [35].

**Figure 7:**
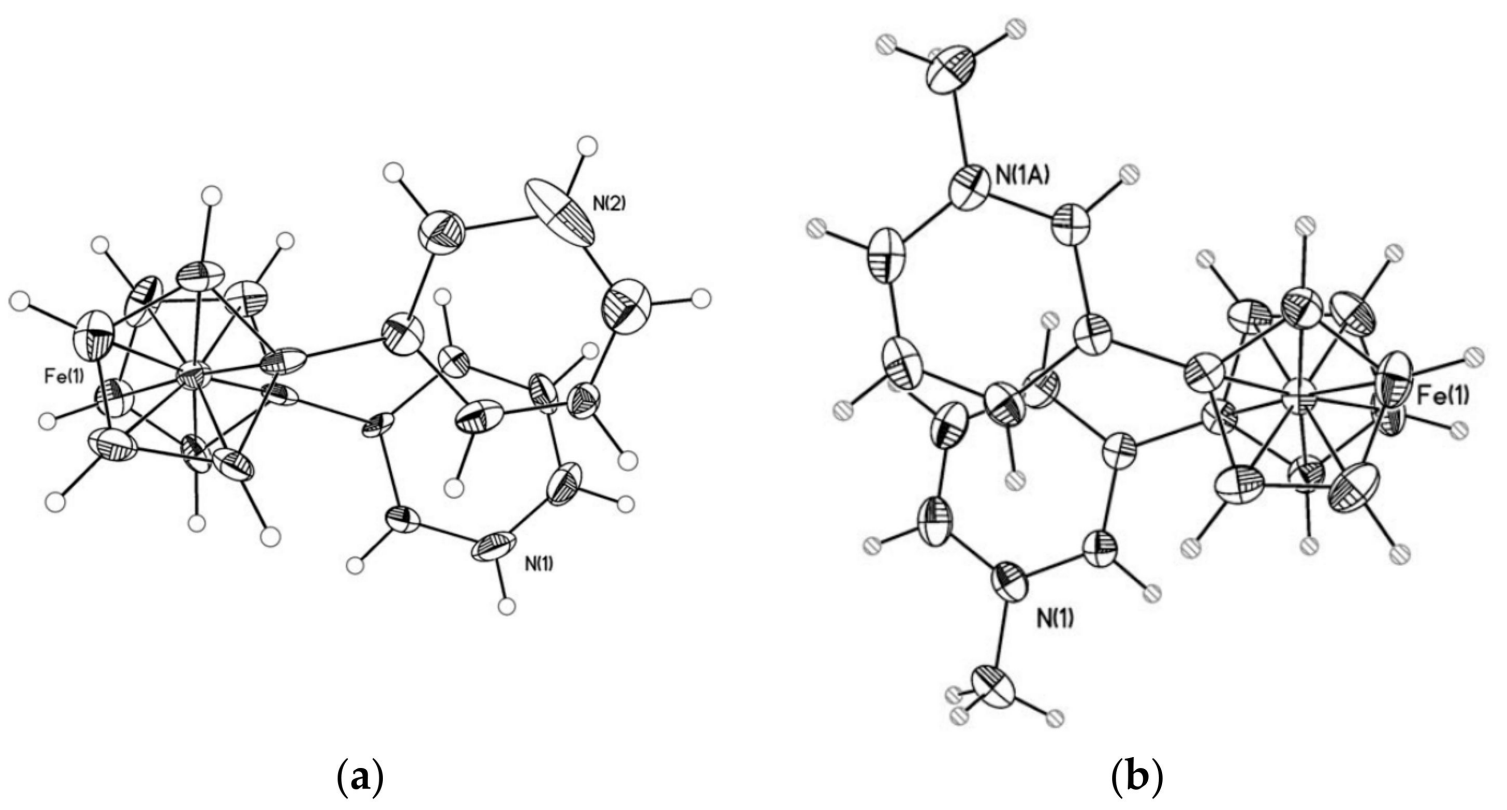
X-ray crystal structures of [(η^5^-3-HNC_5_H_4_-C_5_H_4_)_2_Fe](SbF_6_)_2_ (**a**) and [(η^5^-3-MeNC_5_H_4_-C_5_H_4_)_2_Fe](SbF_6_)_2_ (**b**). Reprinted with permission from [35]. Copyright (2010) John Wiley and Sons.

When 1,1′-bis(pyrid-3-yl)ferrocene was protonated with HSbF_6_, containing weakly coordinating SbF_6_^−^ anion, only a 29.6° rotation of the pyridine “arms” was observed in the solid state [35]. The similar result (rotation degree 37.5°) was found in the case of 1,1′-bis(*N*-methylpyrid-3-inium)ferrocene where methylation of the pyridine substituents was used to introduce the positive charge [35] (Figure 7).

**Figure 8:**
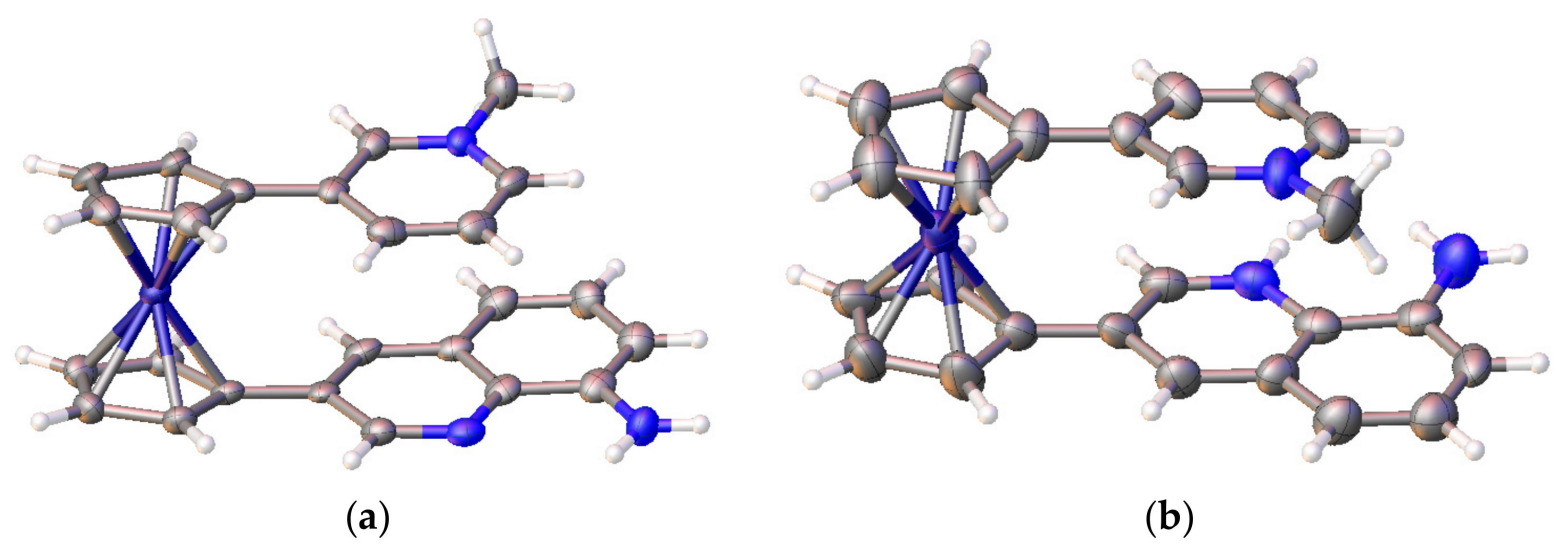
X-ray crystal structures of [(η^5^-3-MeNC_5_H_4_-C_5_H_4_)Fe(η^5^-8-NH_2_-3-NC_7_H_5_-C_5_H_4_)](SbF_6_) (**a**) and [(η^5^-3-MeNC_5_H_4_-C_5_H_4_)Fe(η^5^-8-NH_2_-3-HNC_7_H_5_-C_5_H_4_)](SbF_6_)_2_ (**b**).

The similar result was obtained with an unsymmetrically substituted ferrocene containing a combination of *N*-methylpyridin-3-ium and 8-aminoquinol-3-yl substituents, [(η^5^-3-MeNC_5_H_4_-C_5_H_4_)Fe(η^5^-8-NH_2_-3-NC_7_H_5_-C_5_H_4_)](SbF_6_). The *N*-methylpyridinium group can be regarded as electron-poor, whereas the 8-aminoquinoline group is electron-rich; this together with the greater π-surface of the latter should lead to greater π–π interactions. Indeed, the protonation of [(η^5^-3-MeNC_5_H_4_-C_5_H_4_)Fe(η^5^-8-NH_2_-3-NC_7_H_5_-C_5_H_4_)](SbF_6_) results in negligible rotation of the *Cp* ligands in the solid state (Figure 8), whereas in solution, according to ^1^H-NMR spectral data, the equilibrium is not completely tilted to the π-stacked rotamer [35]. These results indicate that a 1+/1+ charge repulsion is not sufficient to completely overcome the attractive π–π stacking interactions. It is apparent that a higher charge is necessary to overcome the π–π stacking interactions.

### 2.2. Complexation-Driven Molecular Switches

**Scheme 5:**
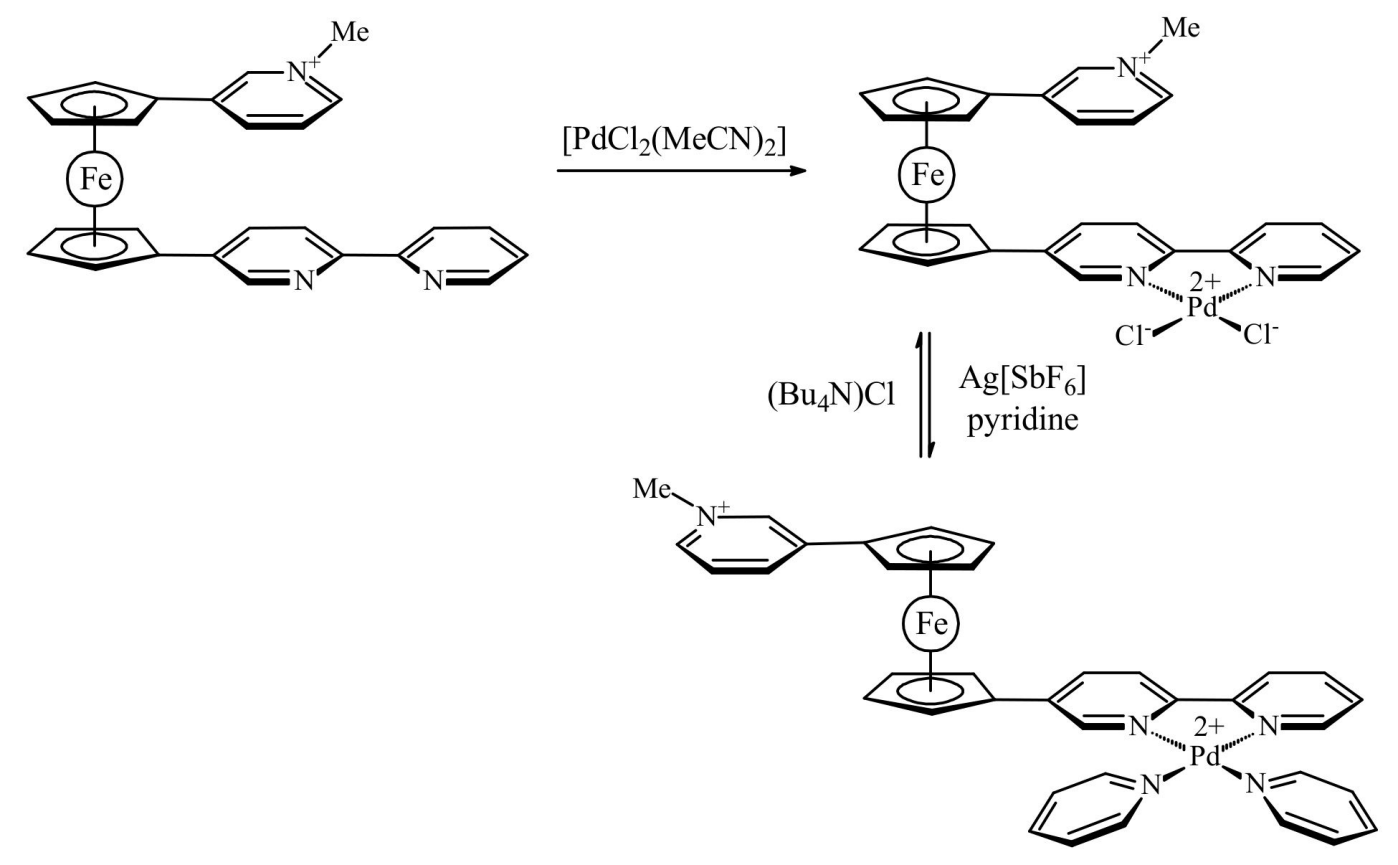
Operation principle of molecular switch based on complexation of π-stacked chelating heteroaromatic substituents.

**Figure 9:**
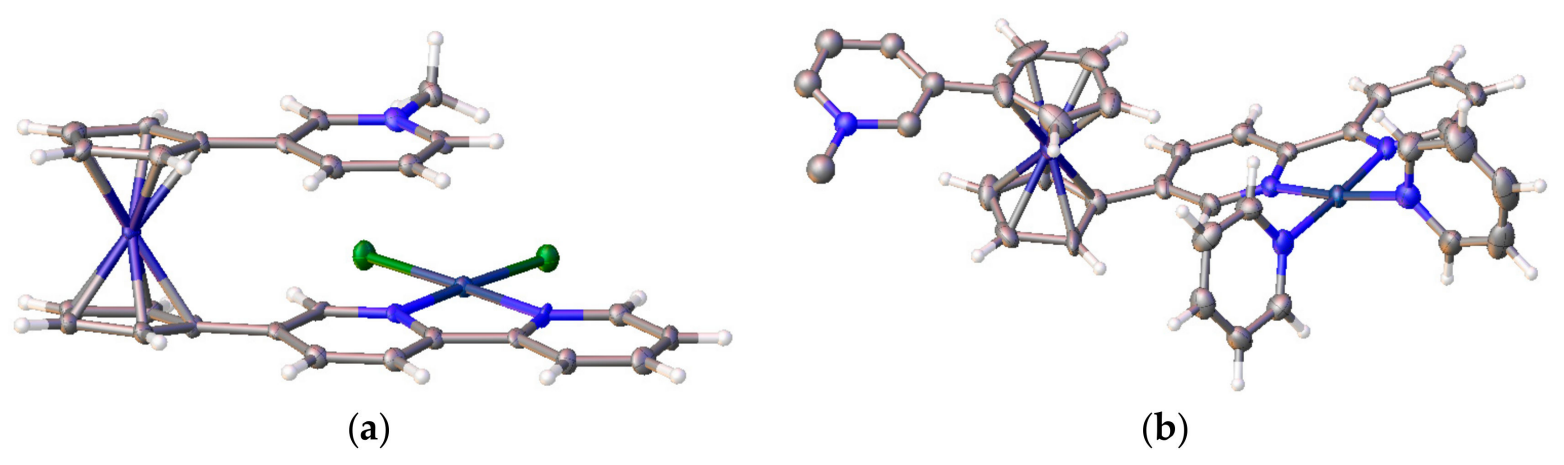
X-ray crystal structures of [(η^5^-3-MeNC_5_H_4_-C_5_H_4_)Fe(η^5^-C_5_H_4_-5-(2,2′-bipy))PdCl_2_]-(SbF_6_) (**a**) and [(η^5^-3-MeNC_5_H_4_-C_5_H_4_)Fe(η^5^-C_5_H_4_-5-(2,2′-bipy))Pd(py)_2_](SbF_6_)_3_ (**b**).

In order to overcome the π–π stacking interactions between the heteroaromatic substituents Bosnich et al. synthesized an asymmetrically substituted ferrocene with a methylated pyridine attached to one *Cp* ring and the 2,2′-bipyridine unit attached to the other, [(η^5^-3-MeNC_5_H_4_-C_5_H_4_)Fe(η^5^-2,2′-bipy-5-C_5_H_4_)](SbF_6_). It was supposed that coordination of transitional metals which are able to form square planar complexes should increase the electrostatic repulsion resulting in the *transoid* conformation. However, the coordination of 2,2′-bipyridine group to palladium(II) dichloride produced only negligible change in the rotation angle (7.43° against 1.44° in ferrocene with uncomplexed bipyridine) (Figure 9a). This small effect can be explained by neutralization of the positive palladium charge by negatively charged chloride ligands that results in the 0/1+ charged compound with π-stacked substituents. The exchange of chloride ligands for neutral pyridines upon treatment with AgSbF_6_ and pyridine results in the 1+/2+ tricationic system. The electrostatic repulsion between the two substituents in this system was found to be sufficient to fully decouple the π-stacking, causing the substituents to rotate almost completely away from one another (the rotation angle ~160°) (Figure 9b). Addition of chloride ions to the system displaces the pyridine ligands regenerating the neutral palladium(II) dichloride complex and restoring the initial π-stacked rotamer (Scheme 5) [35].

**Figure 10:**
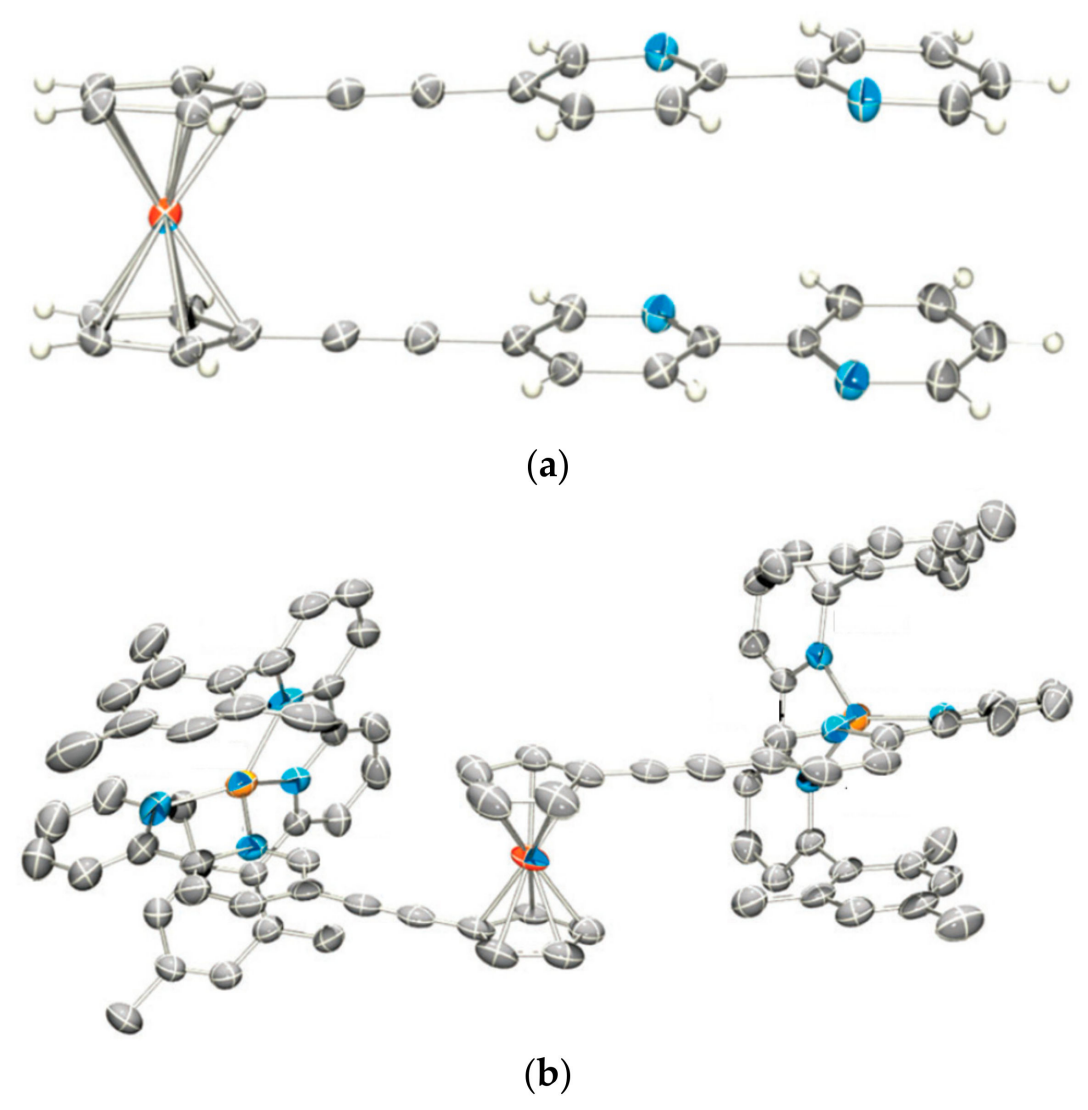
X-ray crystal structures of linkers (η^5^-2,2′-bipy-5-C≡C-C_5_H_4_)_2_Fe (**a**) and [{(6,6′-Mes_2_-2,2′-bipy)Cu}_2_(η^5^-2,2′-bipy-5-C≡C-C_5_H_4_)_2_Fe](PF_6_)_2_ (**b**). Reprinted with permission from [37]. Copyright (2015) The Royal Society of Chemistry (London, UK).

The work by Bosnich has been recently developed further by Crowley and co-workers with the synthesis of ferrocene-based switch with two 2,2′-bipyridine units attached to *Cp* rings via alkyne linkers (η^5^-2,2′-bipy-5-C≡C-C_5_H_4_)_2_Fe [37]. Like the previous system, it relies on the favorable π-stacking between the two substituents to attain the *cis*-conformation (Figure 10a). To achieve the *transoid*-conformation this system requires the complexation with copper(I) along with the sterically bulky 6,6′-dimesityl-2,2′-bipyridine ligand. A combination of electrostatic and steric repulsion destabilizes the *cis*-conformation upon complexation of the {Cu(6,6′-Mes_2_-2,2′-bipy)}^+^ units and lead to the formation of the *transoid*-conformer (Figure 10b) [37].

**Scheme 6:**
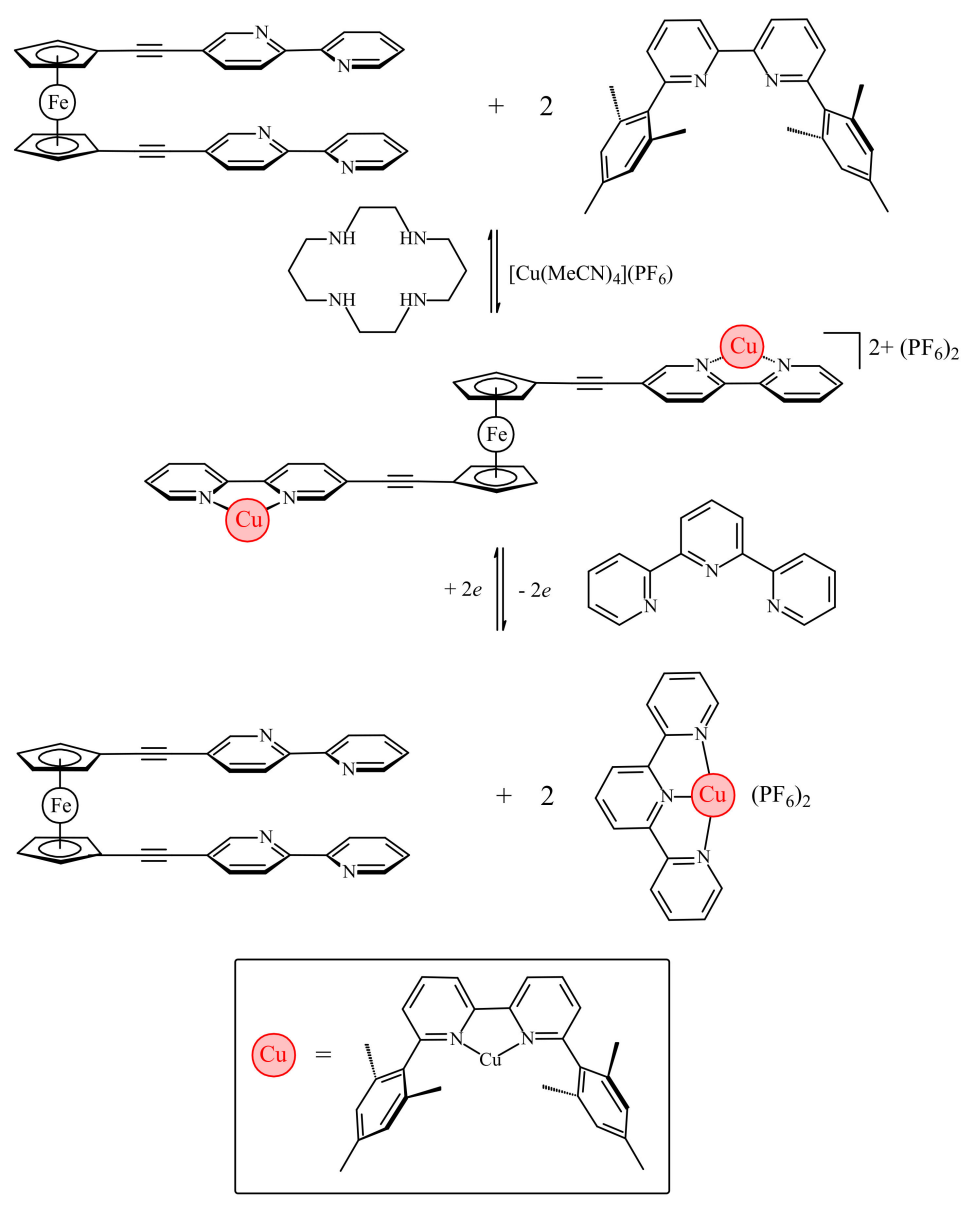
The molecular switch based on complexation of π-stacked chelating heteroaromatic substituents.

The electrochemical switching of the dicopper(I) rotor complex was carried out in the presence of 2,2′:6′,2″-terpyridine ligand (terpy) and oxidation or reduction of the copper ions sets the [(6,6′-Mes_2_-2,2′-bipy)Cu]^n+^ fragments in motion. The oxidation of Cu(I) to Cu(II) results in instability of the former tetrahedral complex and the [(6,6′-Mes_2_-2,2′-bipy)Cu]^2+^ fragments migrate to the terpy ligands generating the more stable five-coordinated [(6,6′-Mes_2_-2,2′-bipy)Cu(terpy)]^2+^ complex and free (η^5^-2,2′-bipy-5-C≡C-C_5_H_4_)_2_Fe. Similarly, the reduction of Cu^2+^ to Cu^+^ results in instability of the [(6,6′-Mes_2_-2,2′-bipy)Cu(terpy)]^+^ complex and the [(6,6′-Mes_2_-2,2′-bipy)Cu]^+^ fragment migrates back to the ferrocene rotor regenerating the [{(6,6′-Mes_2_-2,2′-bipy)Cu}_2_(η^5^-2,2′-bipy-5-C≡C-C_5_H_4_)_2_Fe]^2+^ complex and returning the copper(I) ions to the preferred tetrahedral coordination environment (Scheme 6). The combined ^1^H-NMR and UV-vis spectroscopy study demonstrated that this complexation-driven process could be repeated a number of times (>5) indicating that the switching is completely reversible [37].

**Scheme 7:**
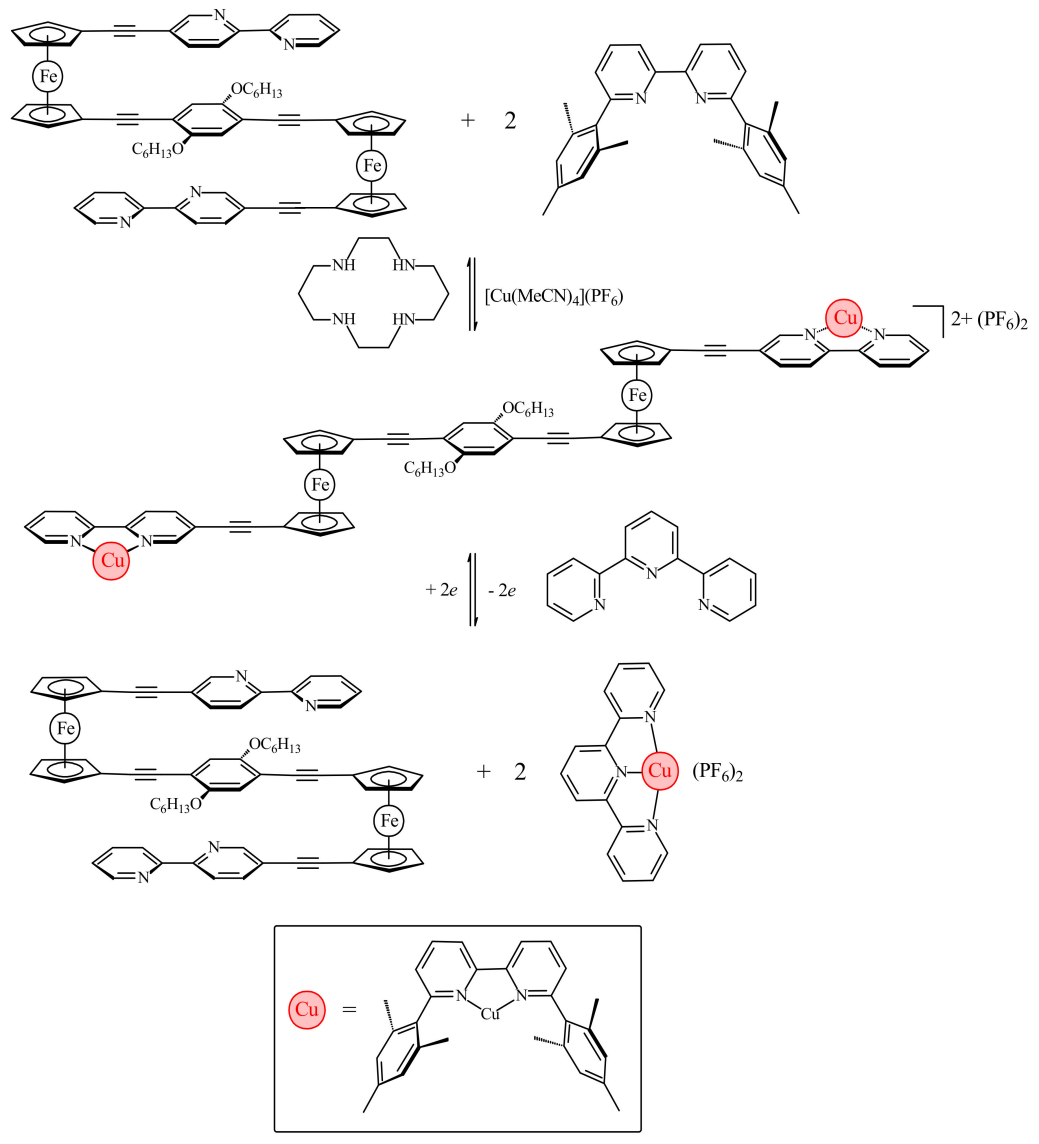
The triple tiered switchable molecular folding ruler based on complexation of π-stacked chelating heteroaromatic substituents.

**Figure 11:**
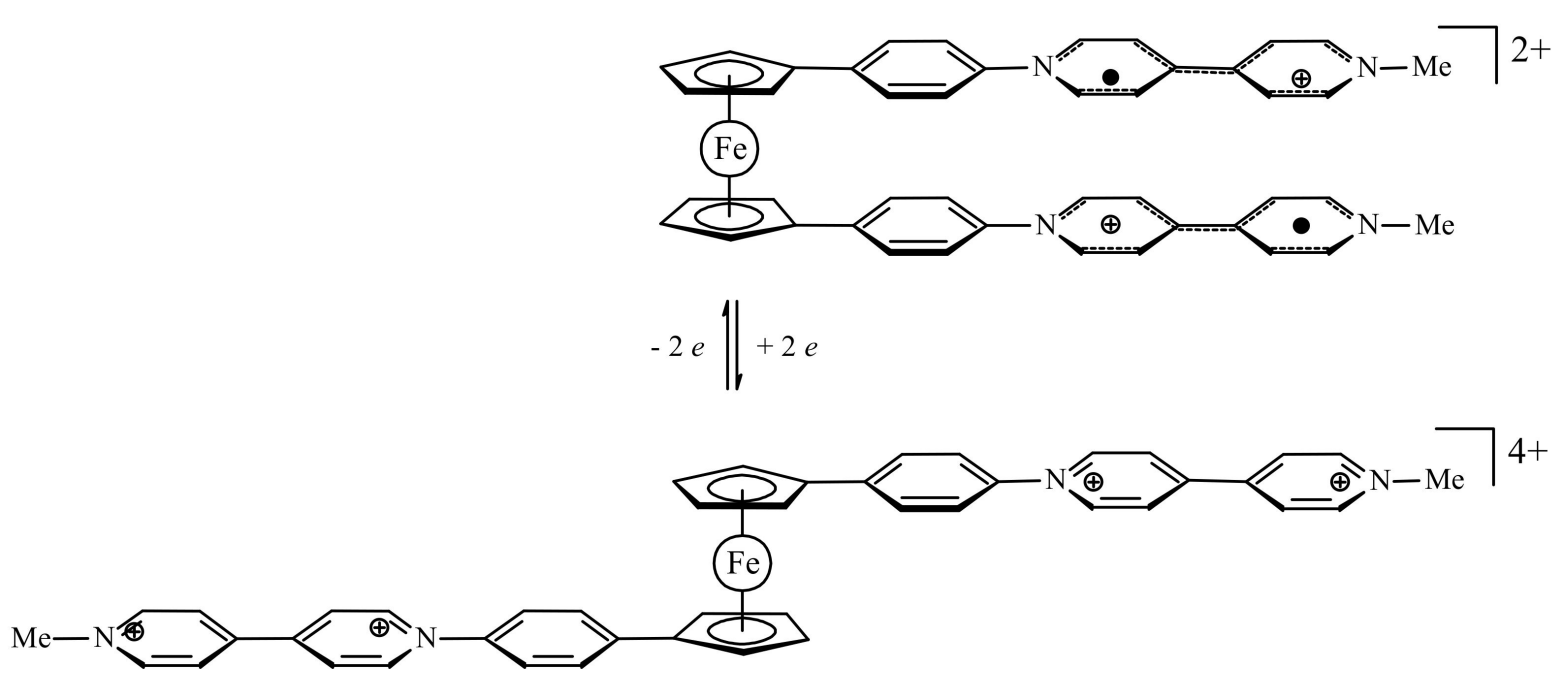

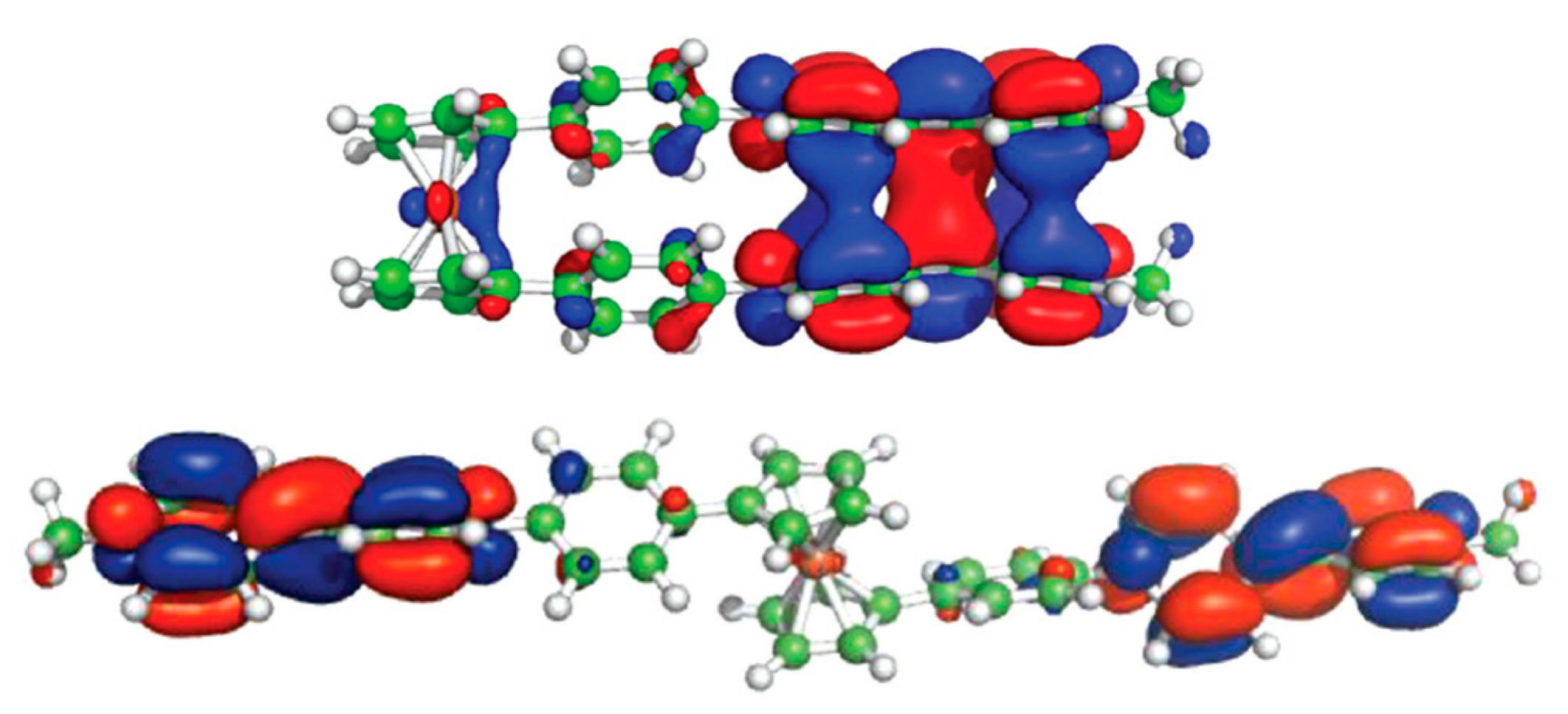
Operation principle (**top**) and calculated frontier molecular orbitals (**bottom**) for molecular switch based on oxidation-reduction of bis(4,4′-bipyridinium) derivative of ferrocene. Reprinted with permission from [40]. Copyright (2012) American Chemical Society.

Sometime later the same approach was used for synthesis of the triple tiered switchable molecular folding ruler 1,4-[(η^5^-2,2′-bipy-5-C≡C-C_5_H_4_)Fe(η^5^-C_5_H_4_-C≡C-)]-2,5-(HexO)_2_-C_6_H_2_, which adopts the stacked conformation both in solution and in the solid state (Figure 11). The copper complexation results in switching from foldered/stacked to extended/unstacked conformation due to electrostatic and steric repulsion (Scheme 7) [38].

**Scheme 8:**
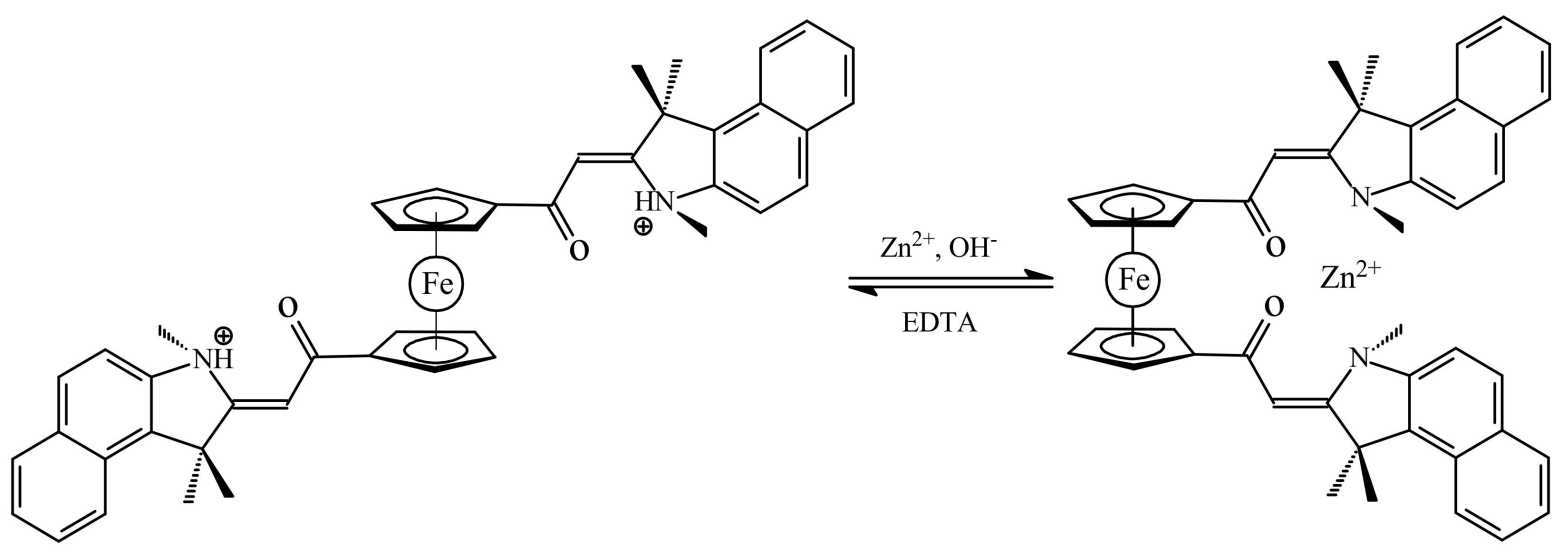
Operation principle of molecular switch based on complexation–protonation of ferrocene with the benzo[*e*]indoline units.

Another example of molecular switch represents ferrocene with the benzo[*e*]indoline units that usually used as an effective fluorescence ligand. When the nitrogen atoms are protonated, the substituents would exhibit repulsion to each other, which could push them onto opposite sides of the switch through the rotation of the ferrocene ligands resulting in the *anti*-conformation. However, the two benzo[*e*]indoline fragments could also complex with metal ions together, and in this way both substituents could be attracted to the same side producing the *syn*-conformation of the switch (Scheme 8). It was found that this switch can serve as a sensor of Zn^2+^ ions. The zinc complexation results in a great chelation-enhanced fluorescence effect whereas other metal ions, such as K^+^, Ca^2+^, Mg^2+^, Ba^2+^, Fe^2+^, Ag^+^, Cu^2+^ and Ni^2+^, produce only negligible effect on the fluorescence. This complexation can be destroyed by addition of a strong chelate agents such as ethylenediamine tetraacetic acid (EDTA) [39].

### 2.3. Electrochemically-Driven Molecular Switches

A model of redox-switchable system based on the bis(4,4′-bipyridinium) derivative of ferrocene was proposed by Bucher et al. Initially, the bis(4,4′-bipyridinium) rotor exists in the *anti*-conformation due to electrostatic repulsion between the substituents. The two-electron reduction results in formation of bipyridinium radicals on each arm of the rotor. These radicals form the intramolecular π-dimer complex in such a way generating the *syn* stacked rotamer. The formation of the intra-molecular π-dimer complex was established by combined UV-vis spectro-electrochemical and ESR study. The DFT calculations at the CAMB3LYP level (6-31* basis set) demonstrated that the LUMO of the open tetracationic species becomes the HOMO of the doubly reduced dicationic species with the effective orbital overlapping being responsible for the efficient association between both radicals giving the closed π-dimer (Figure 11). It should be noted that effective π-interactions are achieved only when rigid conjugated linkers between ferrocene and the 4,4′-bipyridine arms are used [40].

The possibility to use this molecular switch and related systems for nonlinear optic (NLO) materials was sometime later estimated using extended DFT calculations. The rotation of ligands in ferrocene from an “open” *anti*-conformation to a “closed” *syn*-conformation is associated with a remarkably large contrast of nonlinear optical response. The first hyperpolarizabilities of the “open” complexes tend to be zero or very small due to the centrosymmetrical molecular structures, whereas π-dimerized “closed” complexes tend to display large first hyperpolarizabilities due to the donor–π-conjugated bridge–acceptor structures. Thus, these ferrocene-based complexes are able to act as efficient NLO switch [41].

**Scheme 9:**
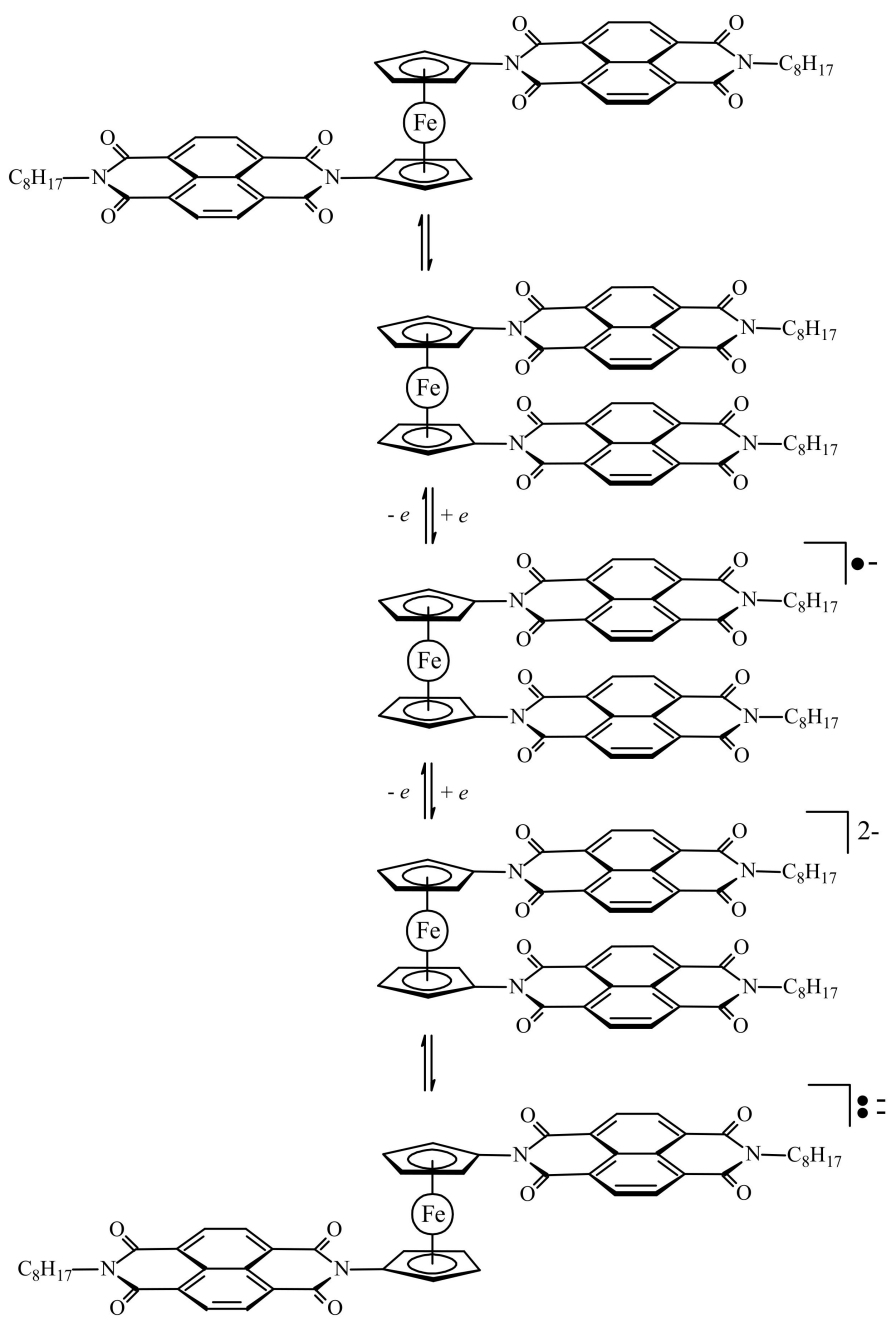
Operation principle of molecular switch based on oxidation-reduction of bis(naphthalenediimide) derivative of ferrocene.

Another model of redox-switchable system where the substituents are naphthalenediimide groups was proposed by Takeuchi et al. (Scheme 9) [42,43].

**Figure 12:**
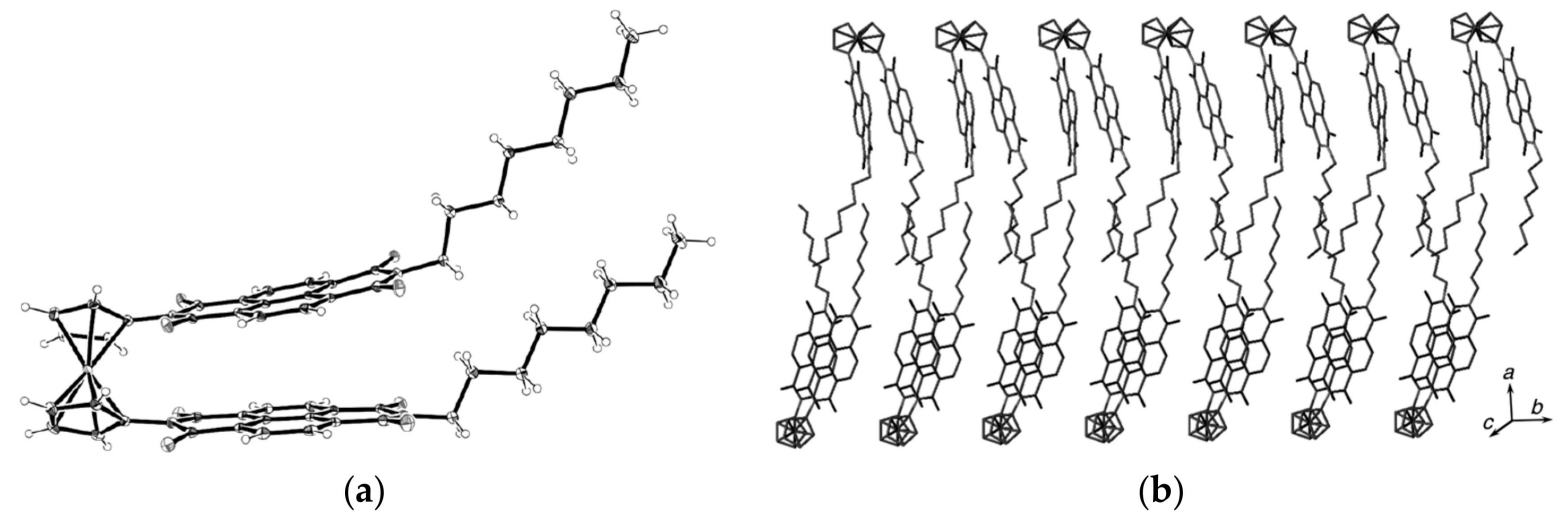
X-ray crystal structure (**a**) and crystal packing (**b**) of the naphthalenediimide derivative (η^5^-Oct-N(CO)_2_-4,5-C_10_H_4_-1,8-(CO)_2_N-C_5_H_4_)_2_Fe. Reprinted with permission from [42]. Copyright (2013) John Wiley and Sons.

In the solid state the neutral switch molecule adopts the π-stacked *syn*-conformation with a dihedral angle of ~9°. However, this stabilization is achieved through not only intermolecular π–π interactions between the naphthalenediimide groups but also intermolecular van der Waals interactions among the octyl groups (Figure 12) [42,43]. The quantum chemical calculations suggest that in solution the open *anti*-conformation is 1.4 kcal/mol more stable than the π-stacked *syn*-conformation. The ^1^H-NMR study indicate rapid interconversion of different conformations at the room temperature and decrease in the rotation rate with decreasing temperature due to the intramolecular interactions between the naphthalenediimide groups [44].

Upon reduction to give the radical anion species, the *syn*-conformation is “locked in” due to the strengthening of the π–π interactions. The second reduction produces the dianionic species that exists in an equilibrium between the extended paramagnetic *anti*- and contracted diamagnetic *syn*-conformations, where the contracted form is preferred due to the pseudo-pairing of the radicals on each substituent (Scheme 9).

**Scheme 10:**
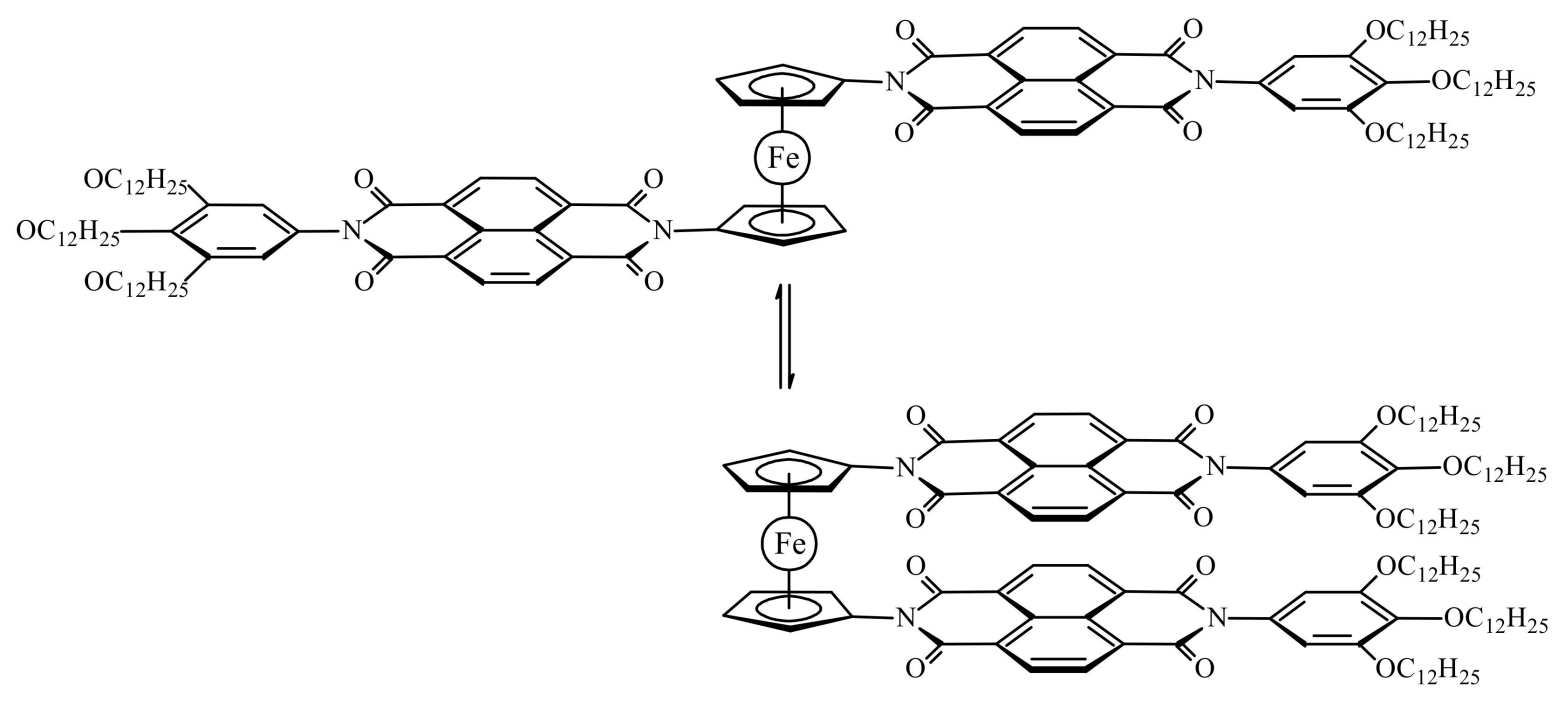
The molecular motor based on oxidation-reduction of bis(naphthalenediimide) derivative of ferrocene.

Based on the ^1^H-NMR spectroscopy data, the similar bis(naphthalenediimide) derivative (Scheme 10) it was found to demonstrate rapid interconversion of different conformations in a solution at the room temperature, whereas the UV-vis absorption, synchrotron X-ray diffraction and AFM studies revealed the formation of fibrous supramolecular assembly in methylcyclohexane and highly concentrated chloroform solutions and film states through π-stacking between the naphthalenediimide units and van der Waals interactions among the long alkyl side chains. The π-molecular switch assembly in the film state exhibits multiple phase transitions associated with conformational changes and intermolecular packing at different temperatures, which are confirmed by differential scanning calorimetry, polarized optical microscopy, and temperature-dependent X-ray diffraction. These thermal transitions also induce changes in the optical and electronic properties, indicating that the conformational flexibility of the π-molecular switch at the molecular level invokes macroscopic dynamic events inside the films [43].

### 2.4. Ferrocene-Based Molecular Scissors and Pliers

**Figure 13:**
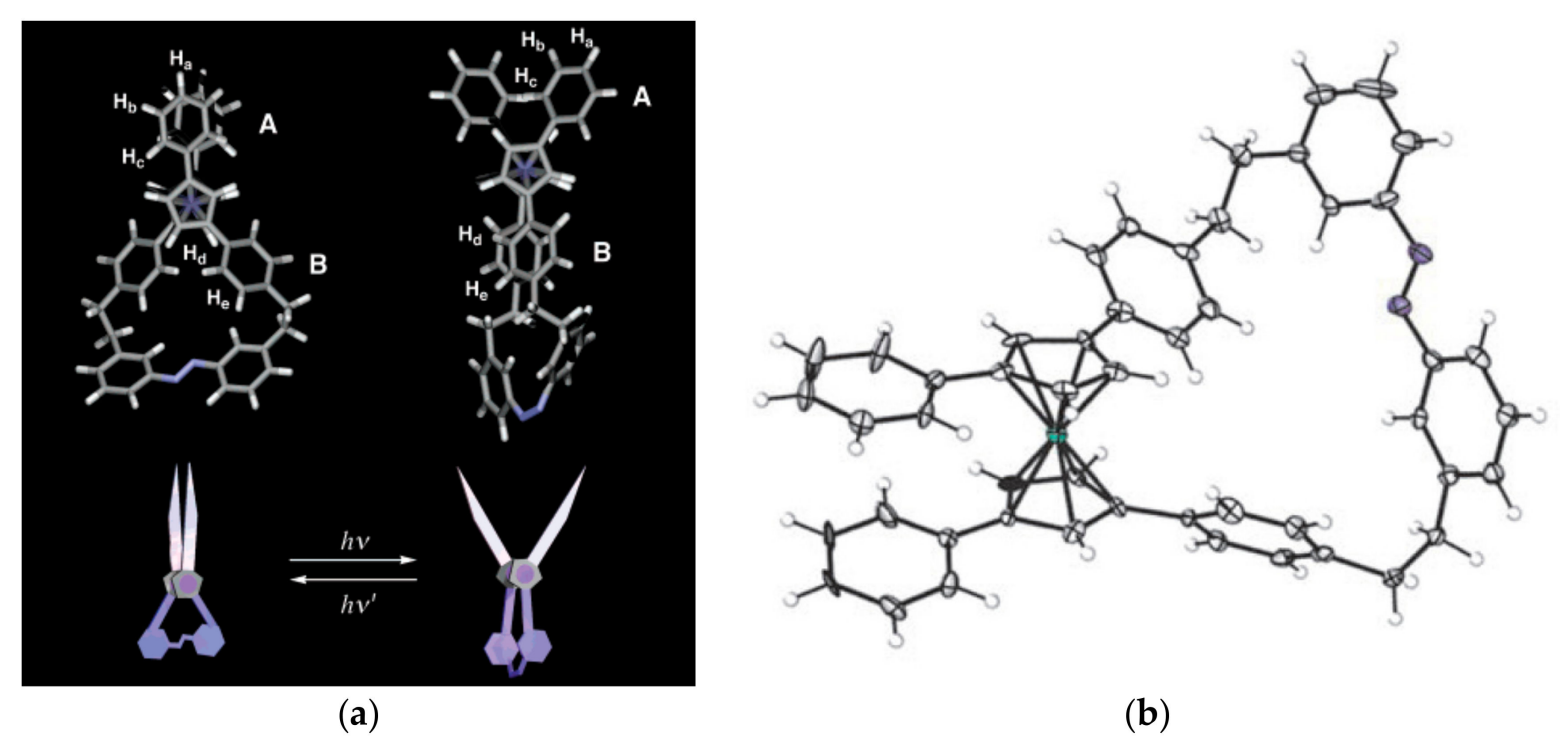
Molecular structures of *trans*- (“closed”) and *cis*- (“open”) isomers of ferrocene-based molecular scissors optimized with DFT calculations (**a**) and X-ray crystal structure of “closed” *trans*-isomer (**a**). Reprinted with permission from [45,46]. Copyright (2003) American Chemical Society and (2007) John Wiley and Sons.

Aida et al. proposed to use the ferrocene unit as a pivot unit to create a range of molecular machines including molecular mimics of macroscopic scissors and pliers [44]. These molecular scissors feature the ferrocene pivot joint, a photoisomerisable azobenzene strap, and two phenyl “blades”. Irradiation of the “closed” *trans*-isomer with UV light (350 nm) causes the azobenzene strap to isomerize and give the *cis*-isomer, where the phenyl “blades” rotate away from one another—a motion reminiscent of scissors opening. Application of visible light (>400 nm) causes photoisomerism back to the “closed” *trans*-form of the scissors (Figure 13) [45,46]. The *trans*-/*cis*-isomer ratio depends on the solvent and the irradiation time. In the dichloromethane solution at the photostationary state the *trans*-/*cis*-isomer ratios were 16/84 and 90/10 under irradiation with UV and visible light, respectively [47].

**Figure 14:**
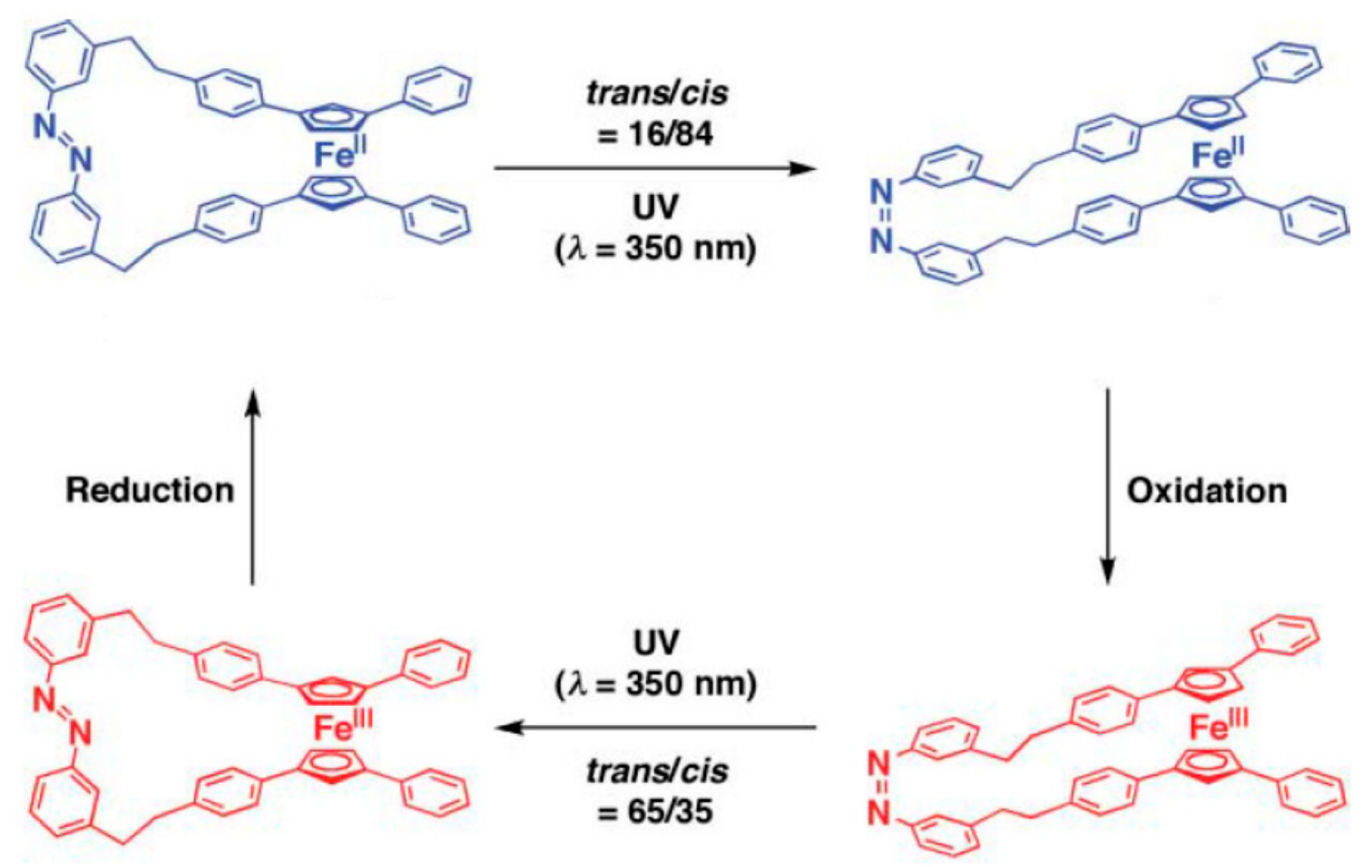
Operation principle of the ferrocene-based molecular scissors by light and redox. Reprinted with permission from [47]. Copyright (2008) The Royal Society of Chemistry.

When the photoisomerized mixture, obtained under irradiation with UV light (*trans*-/*cis*-isomer ratio 16/84), was oxidized by a stoichiometric amount of [(η^5^-C_5_H_4_Cl)_2_Fe]^+^[PF_6_]^−^ and then subjected to further irradiation with UV light, a backward isomerization, in turn, took place to give the oxidized form with *trans*-/*cis*-isomer ratio 65/35. The reduction of this mixture by a stoichiometric amount of Cp*_2_Fe produces neutral scissor with the same *trans*-/*cis*-isomer ratio. Upon exposure to UV light again, the content of *cis*-form increases further to reach 84%, which is identical to that finally attained in the UV induced isomerization of the initial *trans*-isomer (Figure 14) [47].

**Figure 15:**
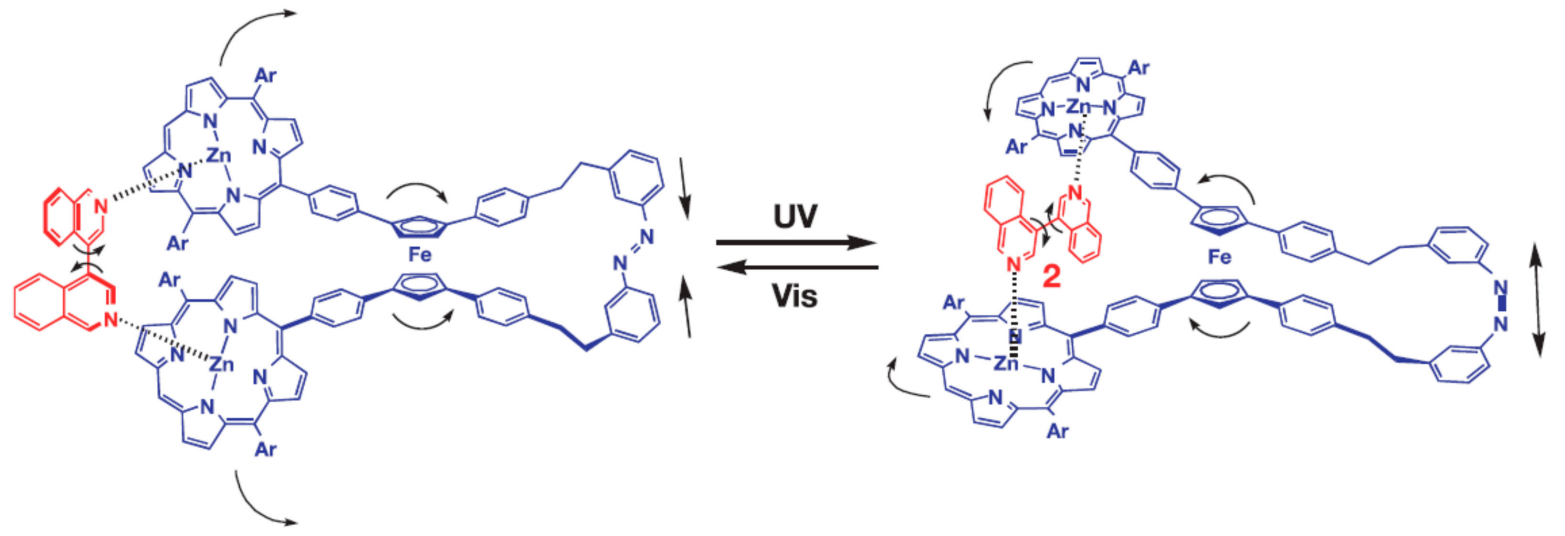
Design and concept of light-powered ferrocene-based molecular pliers. Reprinted with permission from [48]. Copyright (2008) Nature Publishing Group (London, UK).

An advanced version of the light-driven molecular scissors are light-powered molecular pliers that can bind and deform guest molecules. These molecular pliers contain the same ferrocene pivot joint and photoisomerisable azobenzene “handle”, but instead of phenyl “blades” zinc(II)-containing porphyrin units are added to each cyclopentadienyl ring of the ferrocene unit. These pliers can bind bidentate guest molecules, such as 4,4′-biisoqinoline, between the porphyrin units, and the process of opening and closing the pliers acted to distort the bound guest rotamer, adding an additional pedal-like motion to the overall conformational change (Figure 15) [46,48].

It should be noted that, despite the fact that molecular scissors and pliers with azobenzene moiety are impressive models of molecular devices, their efficiency is limited by the incomplete isomerization reaction of the azobenzene moiety.

**Figure 16:**
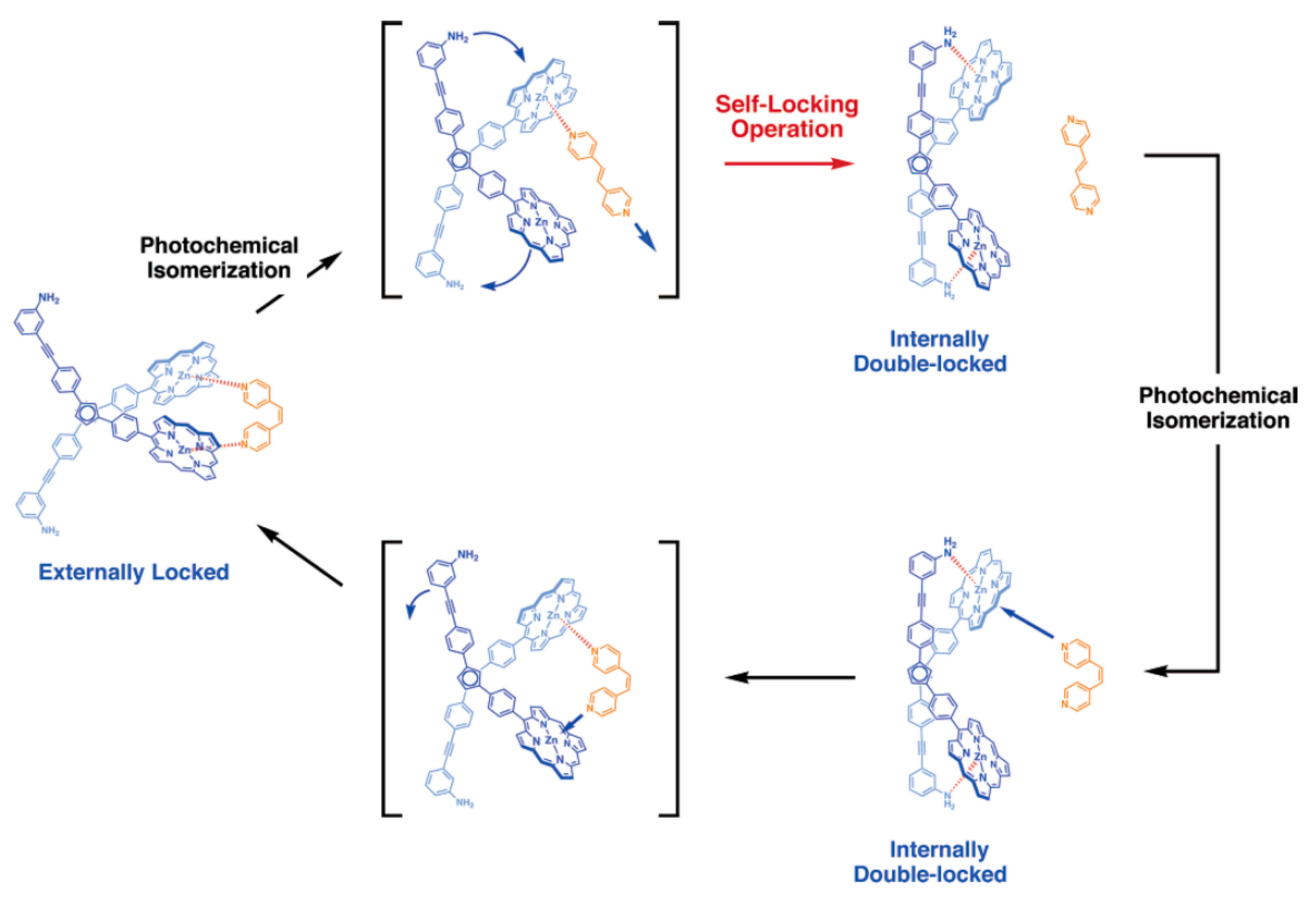
Design and concept of photo-switchable ferrocene-based host–guest system (aryl groups in zinc-porphyrin units are omitted for clarity). Reprinted with permission from [49]. Copyright (2006) American Chemical Society.

The same group designed photo-switchable host–guest system consisting of self-locking ferrocene-based host and 1,2-bispyridylethylene as a photoresponsive key. The host is composed of the ferrocene unit as a rotary module, which bears zinc porphyrin and aniline units at each cyclopentadienyl ring. The rotary motion allows for the aniline group to come closer to the zinc porphyrin units forming two Zn-N coordination bonds simultaneously. Although the interactions between the zinc porphyrins and the anilines is rather weak, such double intramolecular Zn-N coordination is strong enough to lock the rotary motion producing internally double-locked molecule (Figure 16). The photoresponsive key carries two pyridine units capable of coordinating to the zinc porphyrin units in competition with the internal aniline groups. This compound is photochromic, where UV irradiation allows for its *trans*-to-*cis* isomerization, while visible light irradiation of the resulting *cis* form in the presence of triplet sensitizers such as zinc porphyrins gives rise to its backward isomerization. These two isomeric forms have quite different affinities to the host molecule, where the *cis*-form of 1,2-bispyridylethylene is much favored over the *trans*-form. Therefore, the host molecule is kept self-locked in the presence of the *trans*-isomer, however when the *trans*-isomer is photochemicaly isomerized into its *cis*-form the host turns to accommodate resultant *cis*-isomer at its binding site to form the stable host-guest complex having two zinc-pyridyl coordination bonds. Namely, the internal double lock is released, and the host molecule spontaneously transforms into an externally locked state. However, when the *cis*-form in the complex is isomerized into the *trans*-form, the resultant *trans*-isomer detached from the rotary host which spontaneously retrieves the internally double-locked state via rotary motion (Figure 16) [46,49].

**Figure 17:**
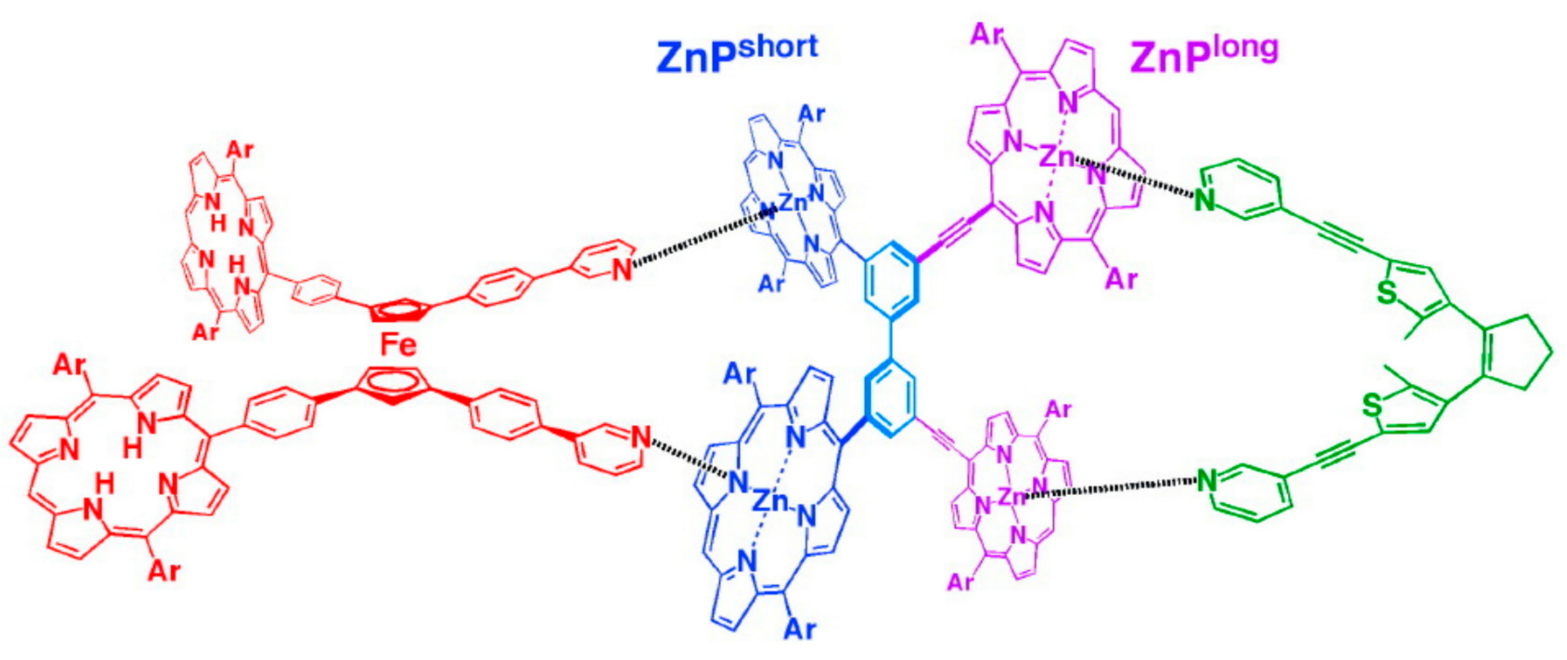
Design of photoactive ternary complex containing ferrocene-based scissors. Reprinted with permission from [50]. Copyright (2008) American Chemical Society.

Sometime later, a ternary complex comprising of the dithienylethene unit as a photochromic component on the one end, a scissoring ferrocene-based component on the other, and a bridging component in the middle was designed by the same group (Figure 17). Upon photoirradiation, the dithienylethene unit undergoes open/closing motion, which gives rise to a rotary motion of the bridging unit and a synchronous scissoring motion of the ferrocene scissors on the other end [50].

**Figure 18:**
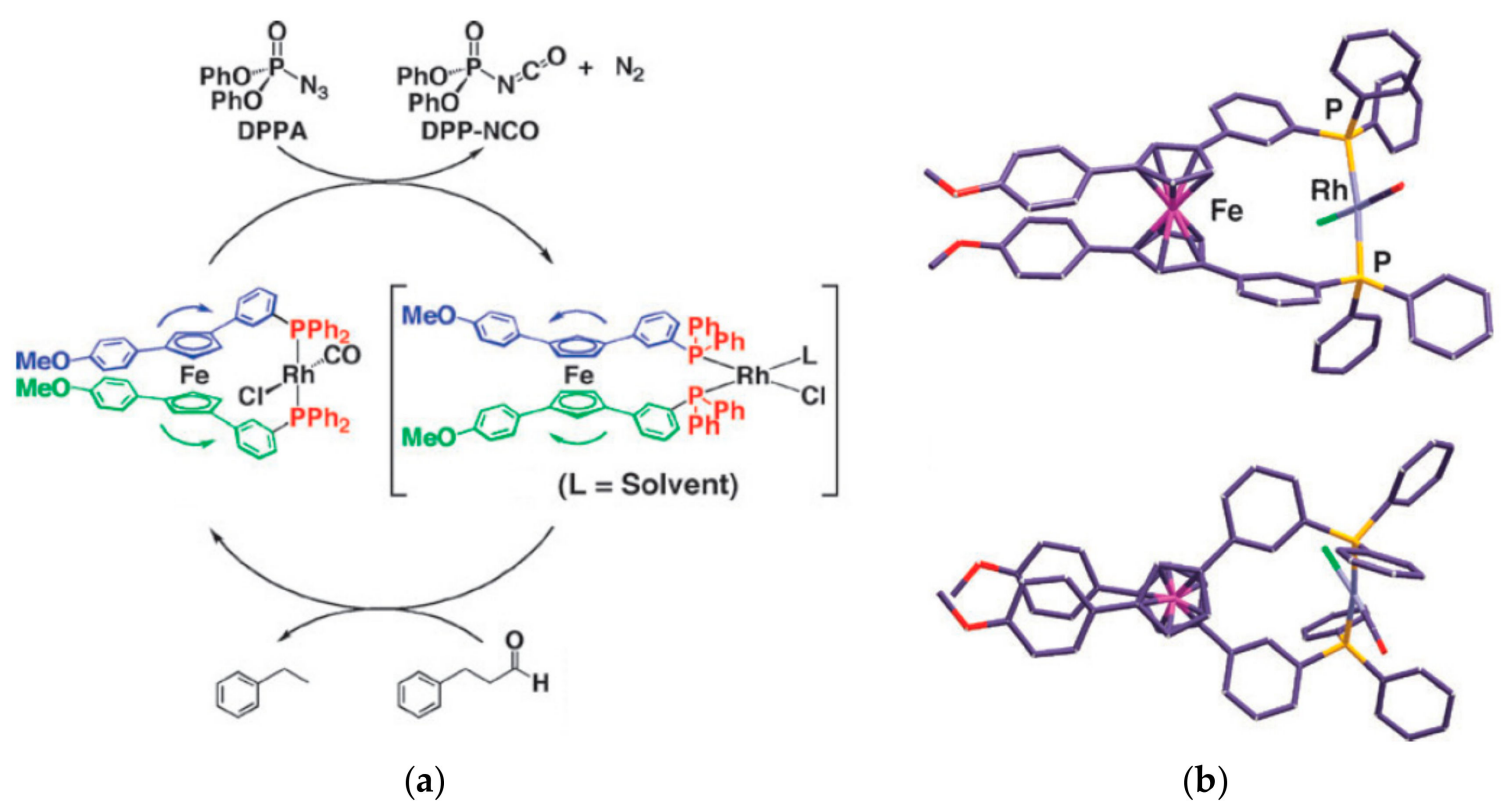
The proposed reaction cycle of the ferrocene-based catalytically driven scissors (**a**) X-ray crystal structure of the scissors with rhodium phosphine complex in the *trans*- (closed) configuration—side view (**top**) and top view (**bottom**) (**b**). Reprinted with permission from [51]. Copyright (2008) The Royal Society of Chemistry.

In another version of the ferrocene-based molecular scissors a Vaska-type rhodium complex and diphenylphosphoryl azide and aldehyde act as an engine and fuel, respectively, for the scissors. The catalytic decarbonylation of aldehyde causes the rhodium complex to interconvert between the *trans*- and *cis*-forms. As a result, the methoxyphenyl “blades” open and close continuously until the reactants/fuel is exhausted (Figure 18) [51].

It should be noted that despite the good potential for 1,1′,3,3′-tetrasubstituted ferrocenes to act as rotary modules for molecular machines, the practical utility is limited due to complexity of their synthesis and the formation of a mixtures of *rac*- and *meso*-forms [52].

**Figure 19:**
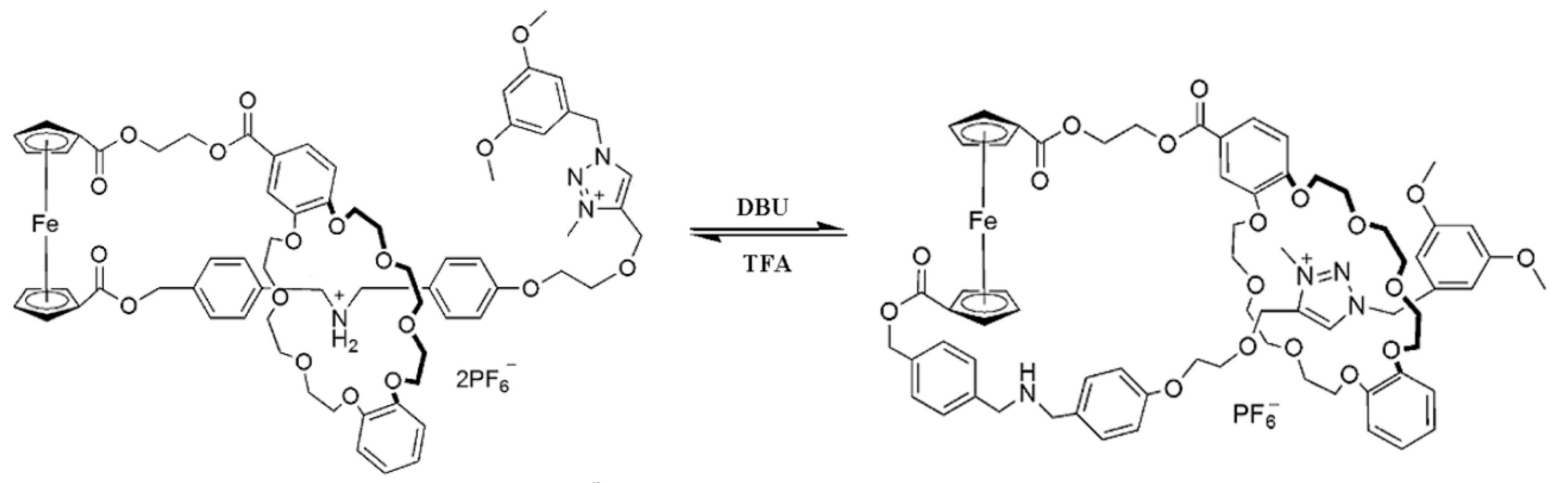
Switching process in bistable ferrocene-based [1]rotoxane. Adopted with permission from [53]. Copyright (2012) American Chemical Society.

The rotatory motion of cyclopentadienyl ligands in ferrocene can be used in the design of other types of molecular switches, for instance the bistable ferrocene-based [1]rotaxane which can switch between two different states caused by chemically driven inclusion and exclusion of an ammonium/amine group by the dibenzo-24-crown-8 (DB24C8) macrocycle. The [1]rotaxane employs the ferrocene unit as an axle with covalently attached the DB24C8 macrocycle and a rod-like thread at each cyclopentadienyl ring, respectively. The rod part which contains two distinguishable recognition sites, namely, a dibenzylammonium site and *N*-methyltriazolium site, was threaded into the macrocycle with a bulky 3,5-dimethoxybenzene stopper situated at the end of the rod part to form an interlocked [1]rotaxane structure. In the original state, the DB24C8 macrocycle encircles the dibenzylammonium cation, which makes the amine group “included”; after deprotonation with a base, the amine group become “excluded” because of its movement and the macrocycle encircles the *N*-methyltriazolium cation (Figure 19) [53].

In general, the main advantages of ferrocene in addition to extremely high stability are its low cost and availability, as well as well-developed methods of functionalization. In addition, aromatic substituents in the ferrocene molecule are located the plane of the cyclopentadienyl rings, which makes it possible to use π-interactions between them to stabilize the *syn*-conformers. However, the stabilization of the *anti*-conformers is mostly based on repulsive interactions between the same-charged substituents, which does not always make it possible to fix clearly the angle of rotation of the ligands when repulsion weakens as the distance between the charges increases. This problem can be solved by introducing a second substituent into the cyclopentadienyl ring, which makes it possible to fix rigidly the angle of rotation of the cyclopentadienyl ligands, as was done by Aida et al. that described synthesis of molecular scissors and molecular pliers which utilize photoisomerization of the bridging azobenzene fragment. Nevertheless, one of the internal drawbacks of this rather effective design is the equilibrium photoisomerization of the azobenzene moiety, which does not allow a complete transformation of one isomer into another, as well as a relatively small angle of rotation of the cyclopentadienyl ligands. Another more common disadvantage is the formation of a mixture of *rac*- and *meso*-diastereomers during synthesis of the corresponding 1,1′,3,3′-tetrasubstituted ferrocenes. The solution of the latter problem requires a more complex design of ferrocene-based molecular switches and, as a sequence, more complicated synthetic schemes for their synthesis.

## 3. Transition Metal Bid(Dicarbollide) Based Molecular Switches

**Figure 20:**
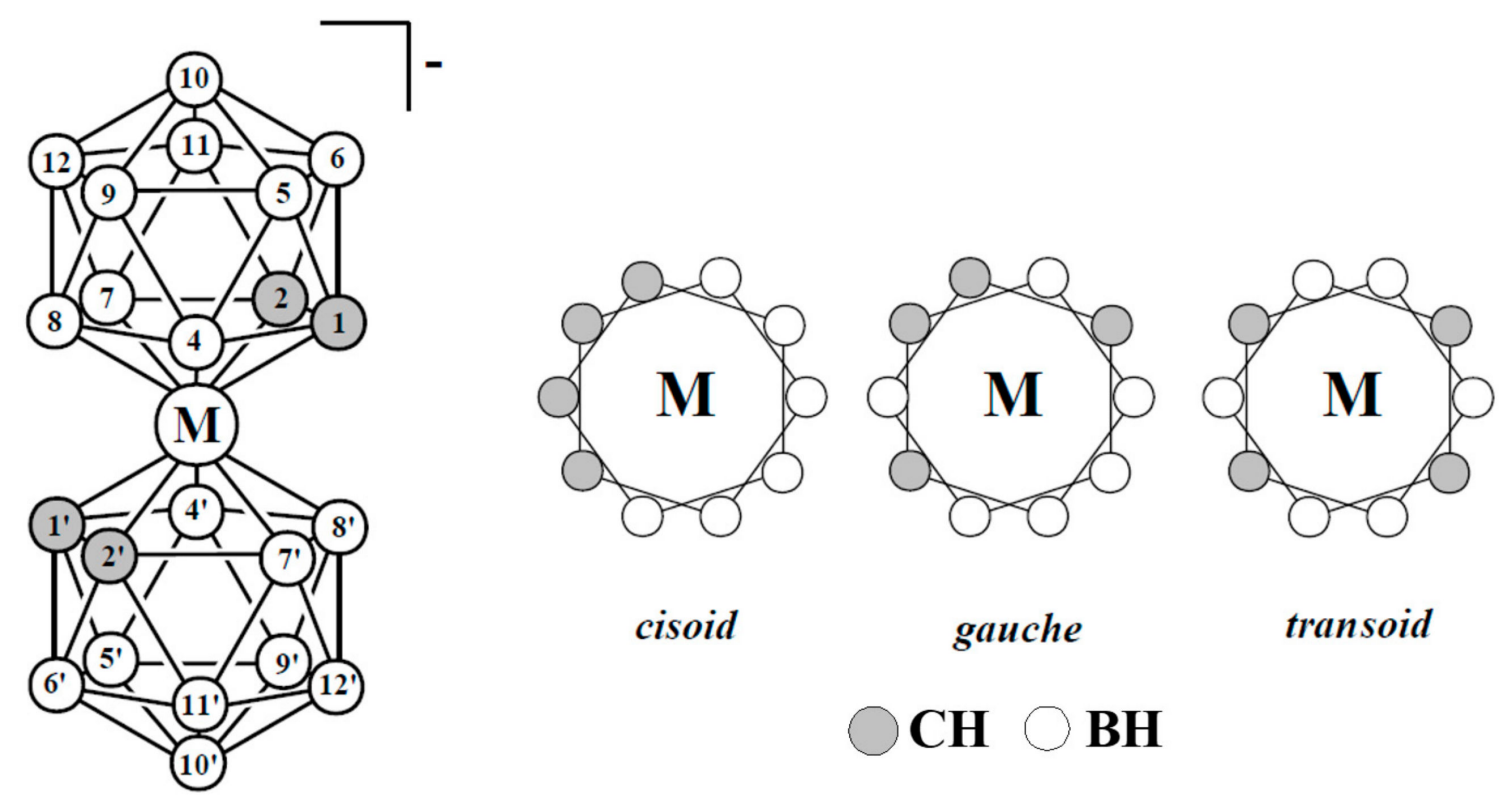
Atom numeration and possible mutual orientation of the dicarbollide ligands in the transition metal bis(1,2-dicarbollide) complexes [3,3′-M(1,2-C_2_B_9_H_11_)_2_]^−^ (M = Fe, Co, Ni).

Other highly stable sandwich complexes that have potential as component of molecular switches are bis(dicarbollide) complexes of transition metals, which are isoelectronic analogs of metallocene (Figure 20). The first synthesis of these complexes has been reported in middle 1960s and their chemical properties are well studied now [54,55,56,57,58]. Unlike cyclopentadienyl ligands, the dicarbollide ligand contain two carbon atoms and three boron atoms in open pentagonal face, that results in energy nonequivalence of different rotamers of bis(dicarbollide) complexes.

**Figure 21:**
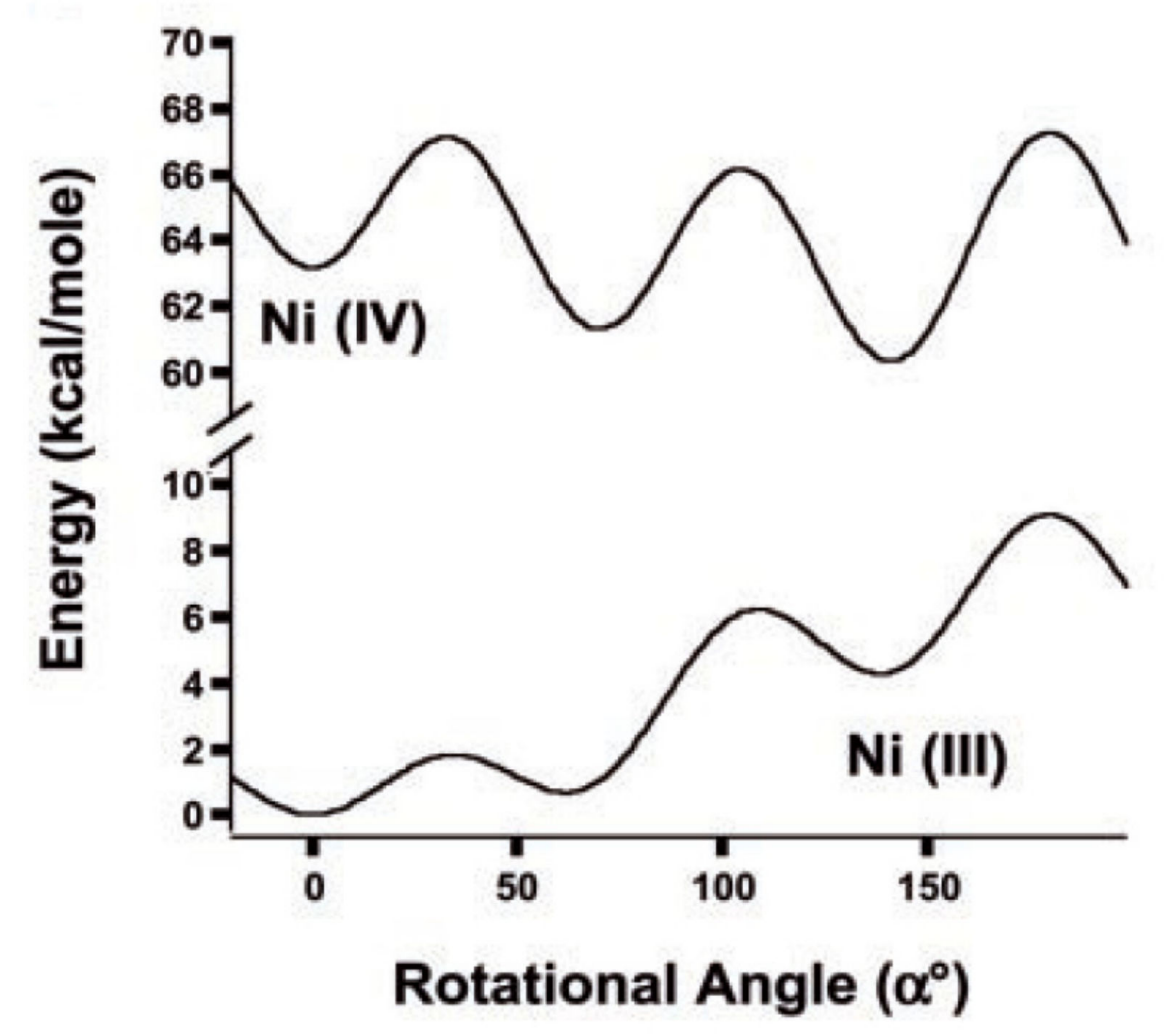
Calculated total energies of nickel(III) bis(dicarbollide) [3,3′-Ni(1,2-C_2_B_9_H_11_)_2_]^−^ (**bottom**) and nickel(IV) bis(dicarbollide) [3,3′-Ni(1,2-C_2_B_9_H_11_)_2_] (**top**) as a function of rotation angle. Adopted with permission from [61]. Copyright (2004) The American Association for the Advancement of Science (Washington, DC, USA).

Preferability of different conformations depend on the nature of the metal and its oxidation state [59,60,61]. Thus, the *transoid* conformation is preferred in the case of nickel(III) bis(dicarbollide), whereas for nickel(IV) bis(dicarbollide) the *cisoid* conformation with the rotation angle about 36° is the most stable (Figure 21) [61]. The high stability of *cisoid* conformation in the nickel(IV) complexes is well illustrated by the fact that in the case of *C*,*C*’-disubstituted dicarbollide ligands the ligand isomerization with migration of one substituted carbon atom from “upper” (closest to the metal atom) to “lower” (farthest from the metal atom) belt takes place instead of turning the dicarbollide ligands to form less sterically hindered *transoid* or *gauche* conformations [62].

**Figure 22:**
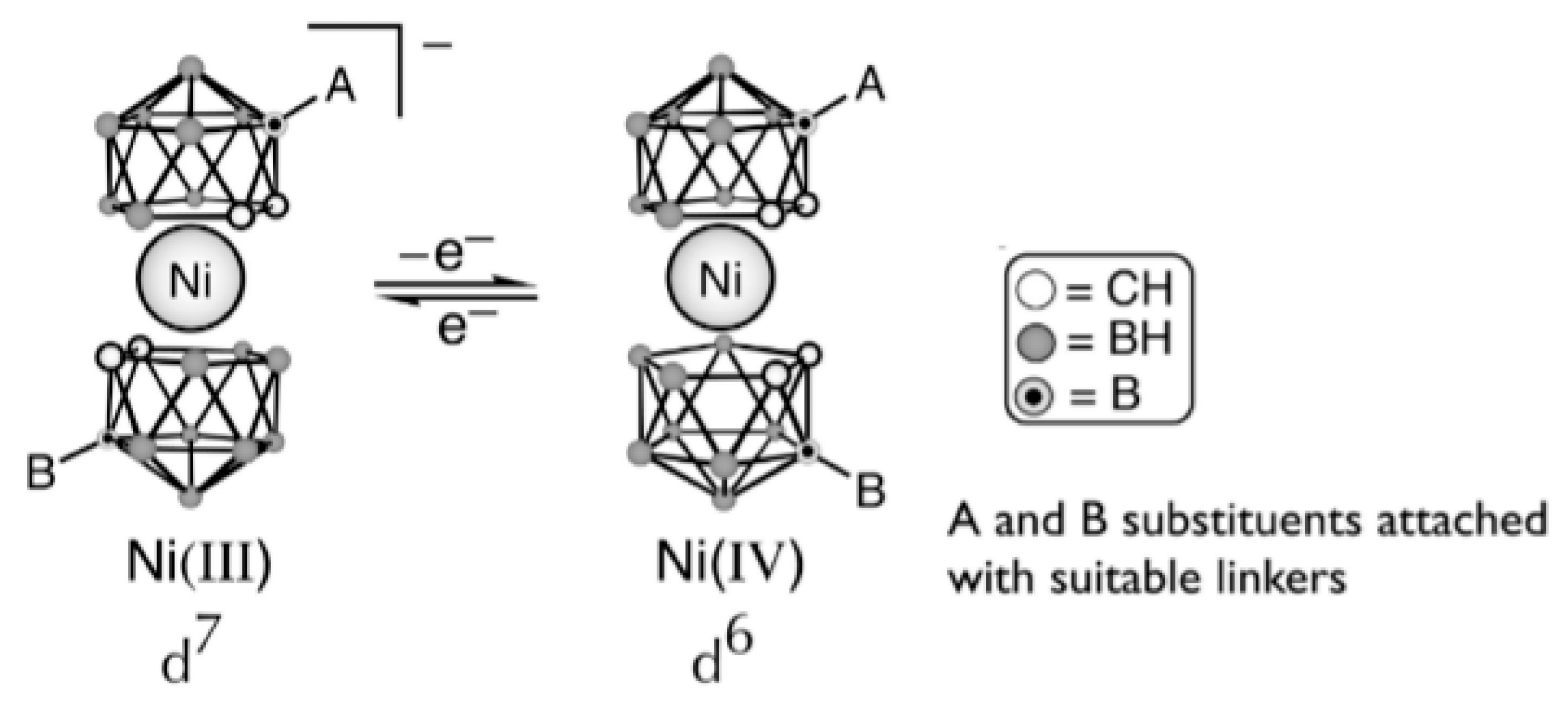
Basic design and concept of electrochemically-driven molecular switches based on nickel bis(dicarbollide). Reprinted with permission from [63]. Copyright (2006) IUPAC (Research Triangle Park, NC, USA).

Hawthorne et al. supposed that the reversible rotational movement of the dicarbollide ligands in nickel bis(dicarbollide) complexes under electrochemical control can be used for development of rotation-based molecular devices and proposed the main principles of the design of such devices [61,63]. In bis(dicarbollide) complexes, substituents in the “upper” belts of dicarbollide ligands do not located in the plane of the open pentagonal face of the carborane ligand, but are directed away from the center of the icosahedron. This, on the one hand, can lead to interactions between substituents in different ligands, and, in particular, excludes the possibility of stabilization of *cisoid*-rotamers due to π-interactions between aryl substituents, as was the case in ferrocene derivatives. On the other hand, the structure of the dicarbollide ligand opens the possibility of synthesis of highly symmetrical 6,6′ (or 8,8′)-di- and 6,6′,8,8′-tetrasubstituted derivatives, which eliminates the problem connected with possibility of formation of diastereomeric mixtures (Figure 22) [63].

**Figure 23:**
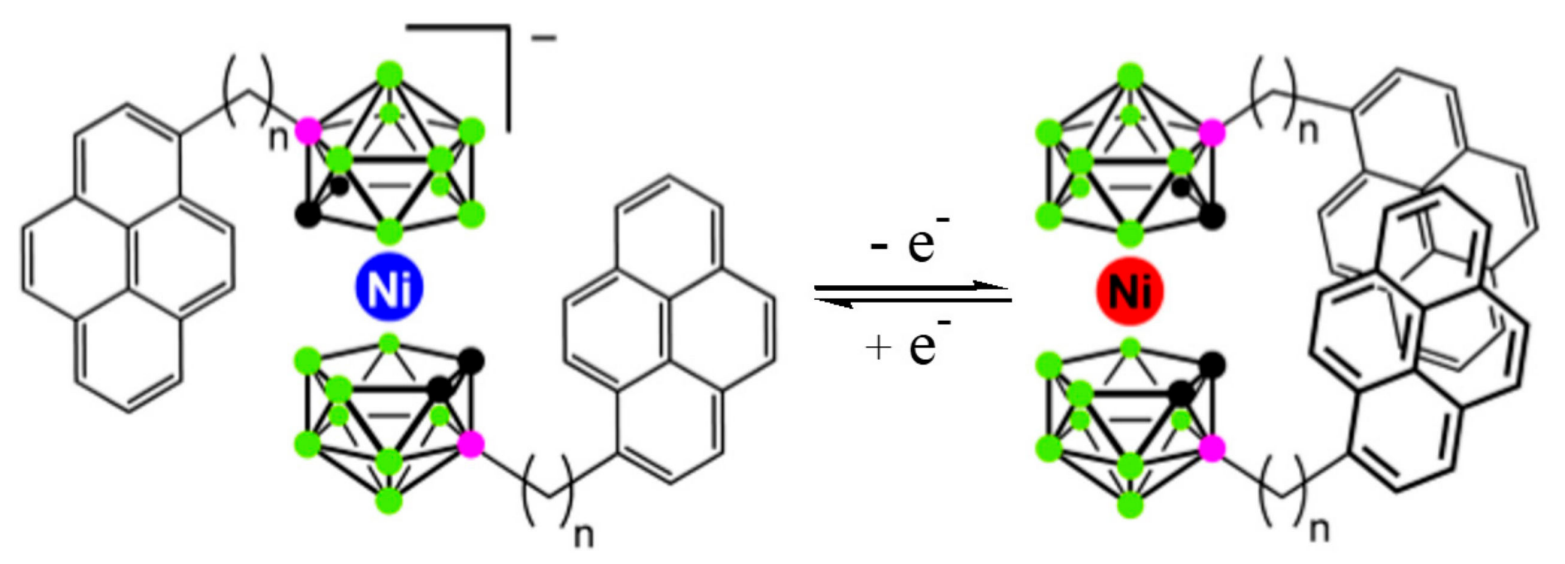
Design and switching process in pyrene-substituted nickel bis(dicarbollides). Adapted with permission from [64]. Copyright (2014) American Chemical Society.

Recently this concept was realized via attachment of pendant pyrene molecules as fluorescent probes (Figure 23). As expected, an intramolecular pyrene excimer emission was observed for the nickel(IV) complex, while the corresponding nickel(III) complex revealed only monomer fluorescence [64].

The calculated energy difference between the most stable *transoid* and the neighboring *gauche* rotamers in the nickel(III) complex was found to be negligible low (~0.1 kcal/mol) therefore the nickel(III) complex exists in solution as a mixture of *transoid* and *gauche* conformers, whereas the nickel(IV) complex due to higher difference in energies of the most stable *cisoid* and the neighboring *gauche* rotamers (~1.2 kcal/mol) in solution exists mainly as the *cisoid* conformer [64].

**Figure 24:**
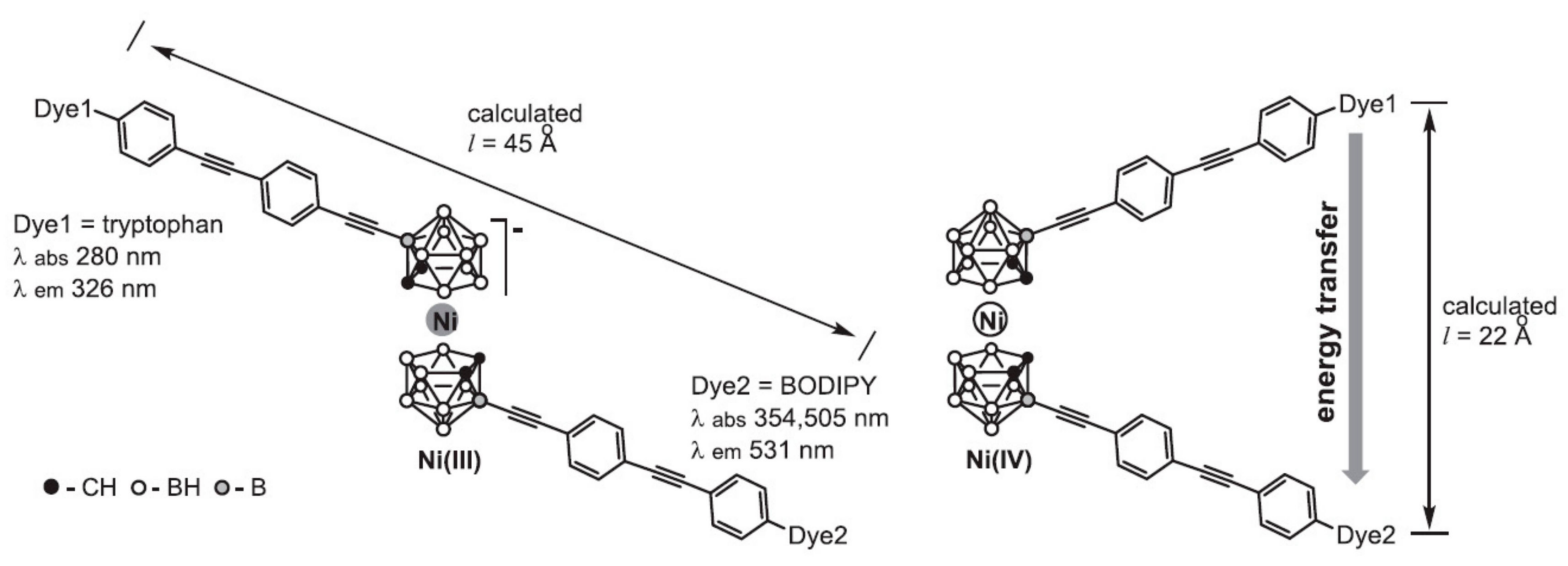
Design of nickel bis(dicarbollide) complexes bearing tryptophan and 4,4-difluoro-4-bora-3*a*,4*a*-diaza-s-indacene (BODIPY) fluorophore molecules. Reproduced with permission from [65]. Copyright (2015) Elsevier.

Sometime later nickel bis(dicarbollide) complex bearing two fluorophore molecules, tryptophan and 4,4-difluoro-4-bora-3*a*,4*a*-diaza-s-indacene (BODIPY), capable of fluorescence resonance energy transfer (FRET) was synthesized. Each fluorophore was connected to the nickelacarborane core by a rigid linker containing two pairs of alternating ethynyl and para-phenylene groups, which created a specific distance (*l*) between the fluorophore molecules depending on the conformation of the nickelacarborane core (Figure 24). The presence (13 Å < *l* < 39 Å) or absence (*l* > 39 Å) of energy transfer in the designed system provides insight into the conformational changes of nickel bis(dicarbollide) in solution. The fluorescence study revealed FRET transfer between the two fluorophores not only for the Ni(IV) complex, but also (to some extent) for the Ni(III) complex, that can be explained by existence at least part of nickel(III) bis(dicarbollide) in solution in *gauche* conformation with *l* < 39 Å [65].

The results obtained demonstrate that transition metal bis(dicarbollides) may provide attractive templates for electrochemically controlled molecular motor/switch-type devices, however due to small energy difference between different rotamers [59,60,61] an additional stabilization of individual rotamers is necessary. As was demonstrated above, in the case of molecular switches based on the nickel bis(dicarbollide) moiety, an additional stabilization of the *transoid* conformation takes on special importance. Such stabilization can be achieved through the introduction of additional substituents on the upper belt of the dicarbollide ligand, that should result in arising weak interactions between the ligands. Some examples of such interactions will be considered below.

**Figure 25:**
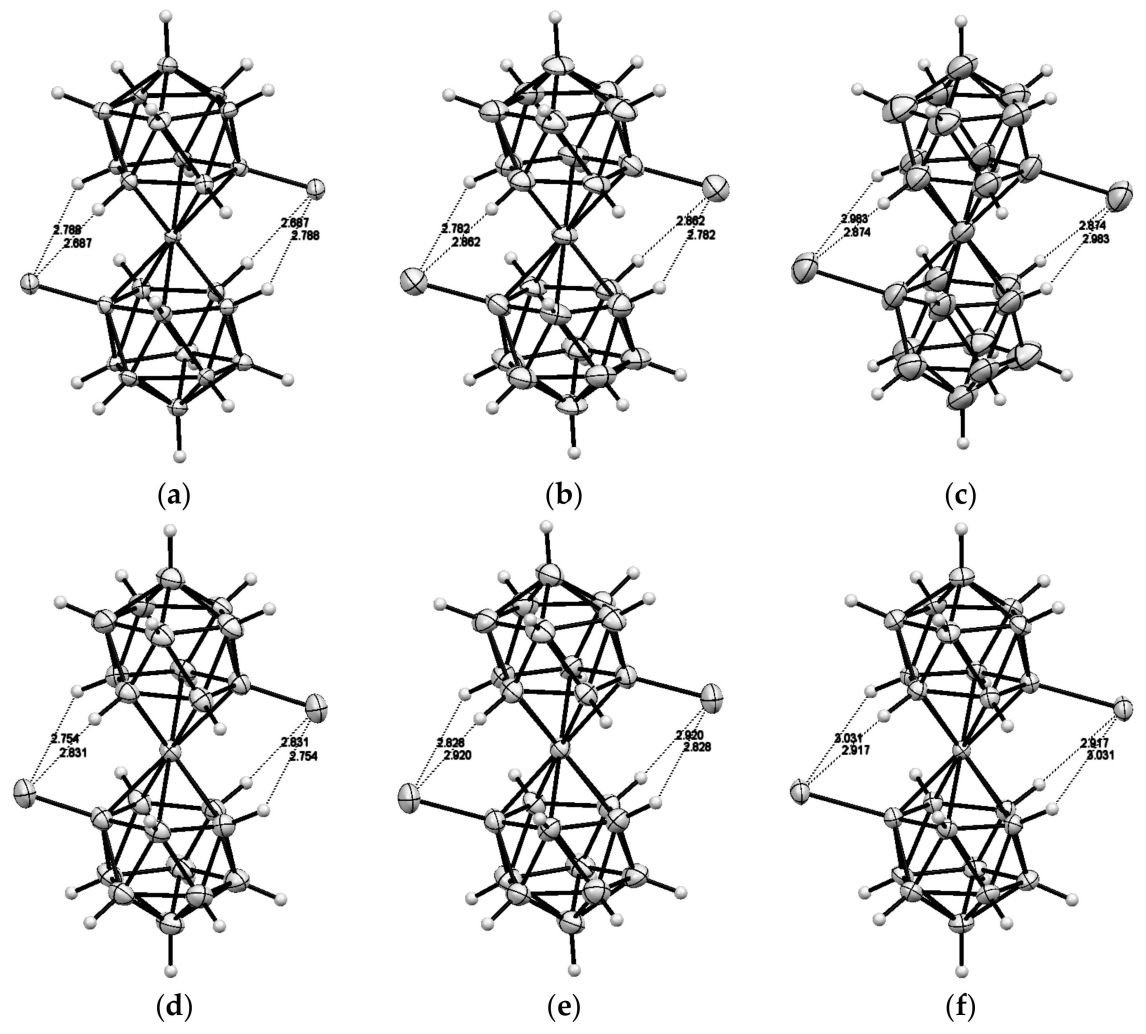
Intramolecular CH…Hal hydrogen bonding in X-ray structures of [8,8′-Cl_2_-3,3′-Co(1,2-C_2_B_9_H_10_)_2_]^−^ [68] (**a**); [8,8′-Br_2_-3,3′-Co(1,2-C_2_B_9_H_10_)_2_]^−^ [69] (**b**); [8,8′-I_2_-3,3′-Co(1,2-C_2_B_9_H_10_)_2_]^−^ [71] (**c**); [8,8′-Cl_2_-3,3′-Fe(1,2-C_2_B_9_H_10_)_2_]^−^ [72] (**d**); [8,8′-Br_2_-3,3′-Fe(1,2-C_2_B_9_H_10_)_2_]^−^ [73] (**e**); [8,8′-I_2_-3,3′-Co(1,2-C_2_B_9_H_10_)_2_]^−^ [73] (**f**).

The simplest type of such interactions is intramolecular CH…Hal hydrogen bonding between slightly acidic metallacarborane CH groups in one ligand and halogen substituents in other ligand. The CH…Hal hydrogen bonds were found to be responsible for stabilization of the *transoid* conformation in the solid-state structures of the 8,8′-dihalogen derivatives of transition metal bis(dicarbollides) [8,8′-X_2_-3,3′-M(1,2-C_2_B_9_H_10_)_2_]^−^ (M = Co, X = Cl [66,67,68], Br [69], I [70,71]; M = Fe, X = Cl [66,72], Br [73], I [73]) (Figure 25). According to Pauling’s principle, the strength of a hydrogen bond should increase with the increase of the electronegativity of the acceptor atom [74], however in this case van der Waals radius of a halogen seems to be more important than its electronegatively. Indeed, no CH…F interactions were found in the 8,8′-difluoro derivative of cobalt bis(dicarbollide) [8,8′-F_2_B-3,3′-Co(1,2-C_2_B_9_H_10_)_2_]^−^ which adopts *cisoid*-conformation [75]. It was found that stabilization of the *transoid* conformation in the 8,8′-dichloro derivative [8,8′-Cl_2_B-3,3′-Co(1,2-C_2_B_9_H_10_)_2_]^−^ in some cases is not enough strong to prevent its transformation to the *gauche* conformation in the solid state [76], whereas *transoid* conformation can be efficiently stabilized through only one pair of the CH…I bonds in the case of the 8-iodo derivative of cobalt bis(dicarbollide) [8-I-3,3′-Co(1,2-C_2_B_9_H_10_)(1′,2′-C_2_B_9_H_11_)]^−^ [77,78]. Additional evidence of the prevailing role of van der Waals radius over electronegatively of a substituent was demonstrated by the absence of intramolecular hydrogen bonding in the 8-hydroxy derivative [8-HO-3,3′-Co(1,2-C_2_B_9_H_10_)(1′,2′-C_2_B_9_H_11_)]^−^ [79] despite the fact that the hydroxy group is the much stronger H-bonding acceptor. The recent study on transmembrane translocation of halogenated derivatives of cobalt bis(dicarbollide) suggests that the *transoid* conformation for [8,8′-X_2_-3,3′-Co(1,2-C_2_B_9_H_10_)_2_]^−^ (X = Cl, Br, I) is kept in solution as well [80].

**Figure 26:**
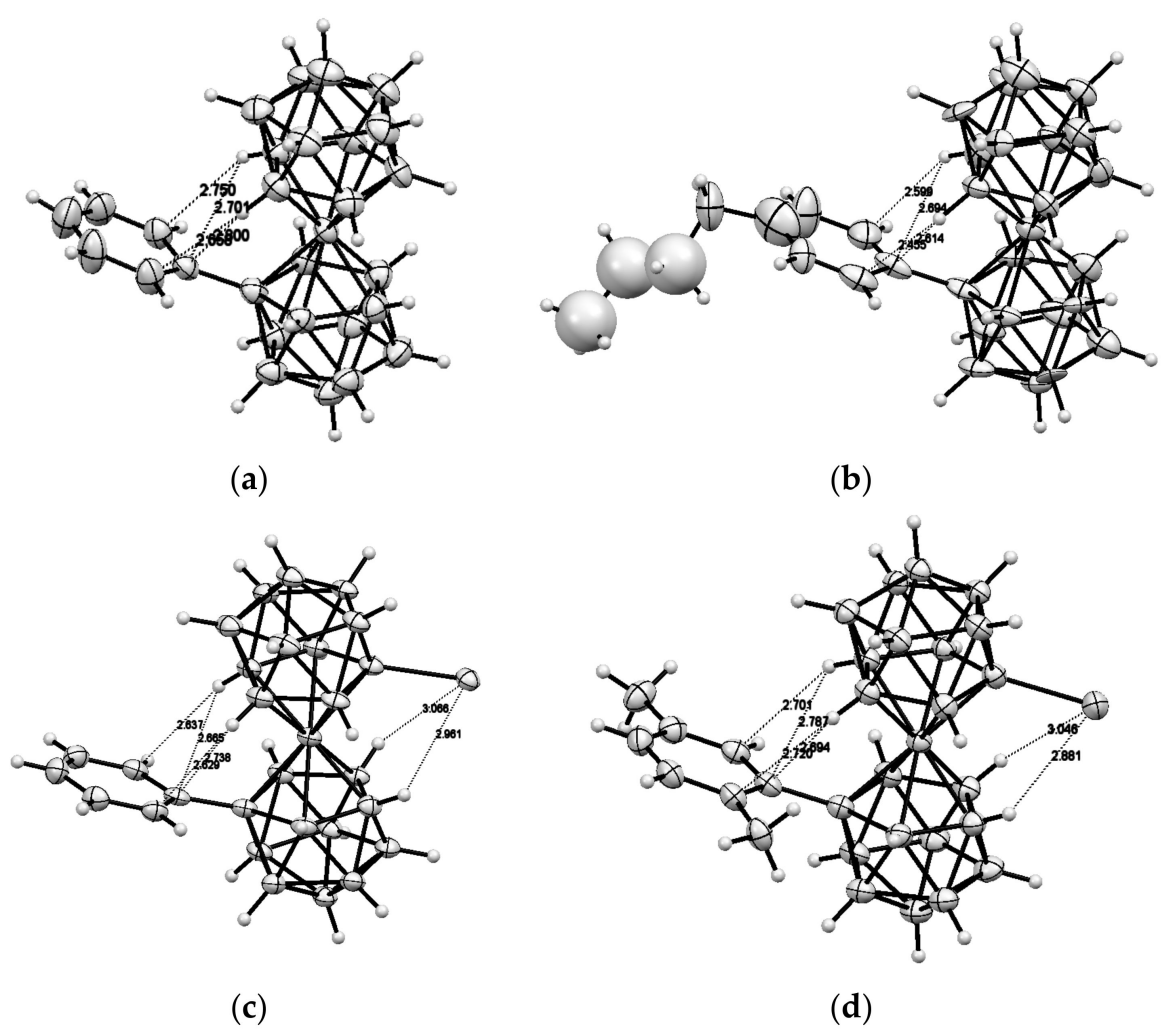
Intramolecular CH_carb_…π aromatic hydrogen bonding in [8-Ph-3,3′-Co(1,2-C_2_B_9_H_10_)- (1′,2′-C_2_B_9_H_11_)]^−^ [81] (**a**); [8-(4-BuC_6_H_4_)-3,3′-Co(1,2-C_2_B_9_H_10_)(1′,2′-C_2_B_9_H_11_)]^−^ [82] (**b**); [8-Ph-8′-I-3,3′- Co(1,2-C_2_B_9_H_10_)_2_]^−^ [83] (**c**); and [8-(2,5-Me_2_C_6_H_3_)-8′-I-3,3′-Co(1,2-C_2_B_9_H_10_)_2_]^−^ [84] (**d**).

Another type of rotamer stabilization was found in the solid-state structures of aryl derivatives of cobalt bis(dicarbollide) [8-Ar-3,3′-Co(1,2-C_2_B_9_H_10_)(1′,2′-C_2_B_9_H_11_)]^−^ (Ar = C_6_H_5_, C_6_H_4_-4-Bu), where stabilization of the *transoid* conformation proceeds through formation of intramolecular aromatic CH_carb_…π bonds between acidic CH groups in one dicarbollide ligand and phenyl substituents in other one [81,82] (Figure 26). Some later derivatives with the “double-locked” *transoid* conformation due to the intramolecular CH_carb_…IB bonds and aromatic CH_carb_…π hydrogen bonds between the dicarbollide ligands [8-Ar-8′-I-3,3′-Co(1,2-C_2_B_9_H_10_)_2_]^−^ were described [83,84] (Figure 26). It was demonstrated that the intramolecular aromatic CH_carb_…π hydrogen bonds exist not only in the solid state but in solution as well [84].

**Figure 27:**
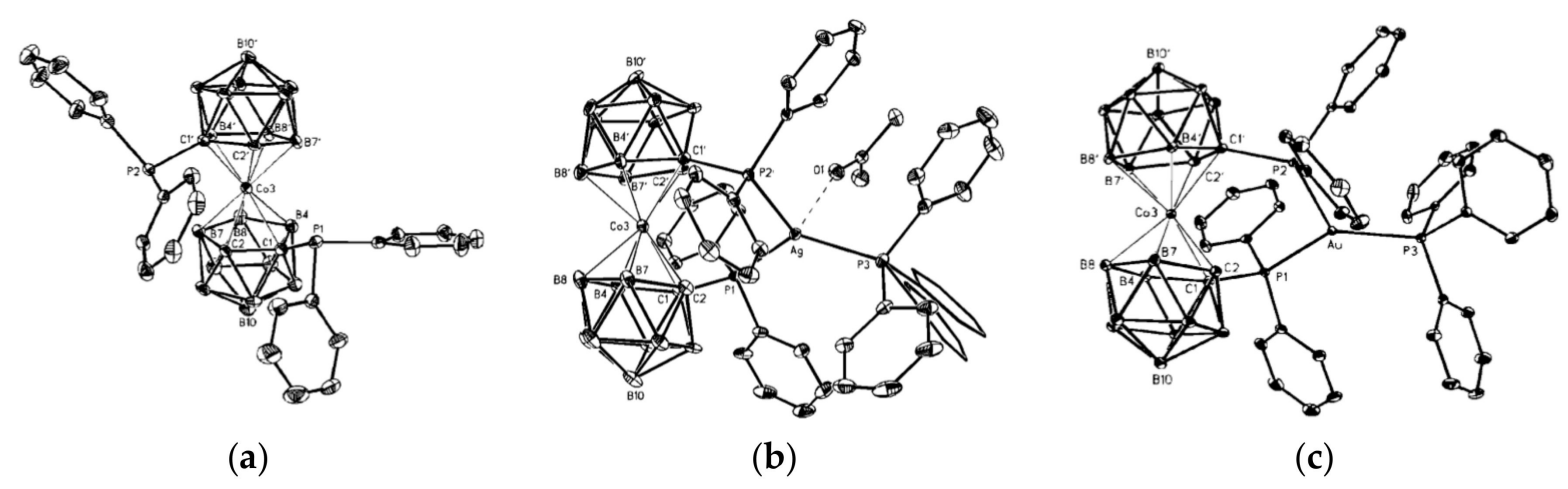
X-ray crystal structures of cobalt bis(dicarbollide) based diphosphine ligand [1,1′-(Ph_2_P)_2_-3,3′-Co(1,2-C_2_B_9_H_10_)_2_]^−^ (**a**) and its silver (**b**) and gold complexes (**c**). Reprinted with permission from [85]. Copyright (2004) John Wiley and Sons.

The results above demonstrate that the intramolecular hydrogen bonding between the dicarbollide ligands can serve as efficient tool for stabilization of individual rotamers of different transition metal bis(dicarbollide) complexes. We suggest that bistability of the cobalt and iron bis(dicarbollide) systems can be achieved through the introduction of substituents that able to form rather weak intramolecular hydrogen bonds between the dicarbollide ligands and stronger dative bonds with external transition metals. In this case, the external metal complexation will stabilize the *cisoid* conformation, whereas the stabilization of the *transoid* conformation will be caused by intramolecular hydrogen bonding between the dicarbollide ligands. Such complexes could serve as the basic structural units for the design of molecular switches, for which the conversion from one form to another would be triggered by cations of external transition metals. To the best of our knowledge, only a few examples of the complexation of external metals with cobalt bis(dicarbollide) derivatives are known. They include silver and gold bis(phosphine) complexes of [1,1′-(Ph_2_P)_2_-3,3′-Co(1,2-C_2_B_9_H_10_)_2_]^−^ [85] and sodium complexes of the thiacrowns [1,1′-μ-{S(CH_2_CH_2_O)_3_CH_2_CH_2_S}-3,3′-Co(1,2-C_2_B_9_H_10_)_2_]^−^ [86] and [8,8′-μ-C_6_H_4_-1,2- (SCH_2_CH_2_OCH_2_CH_2_O)-3,3′-Co(1,2-C_2_B_9_H_10_)_2_]^−^ [87]. However, the complexation produces only small rotations of the dicarbollide ligands in the bis(phosphine) complexes (24.7 and 47.9° for the silver and gold complexes, respectively (Figure 27), whereas conformations of the metal-free thiacrowns have not been determined.

**Figure 28:**
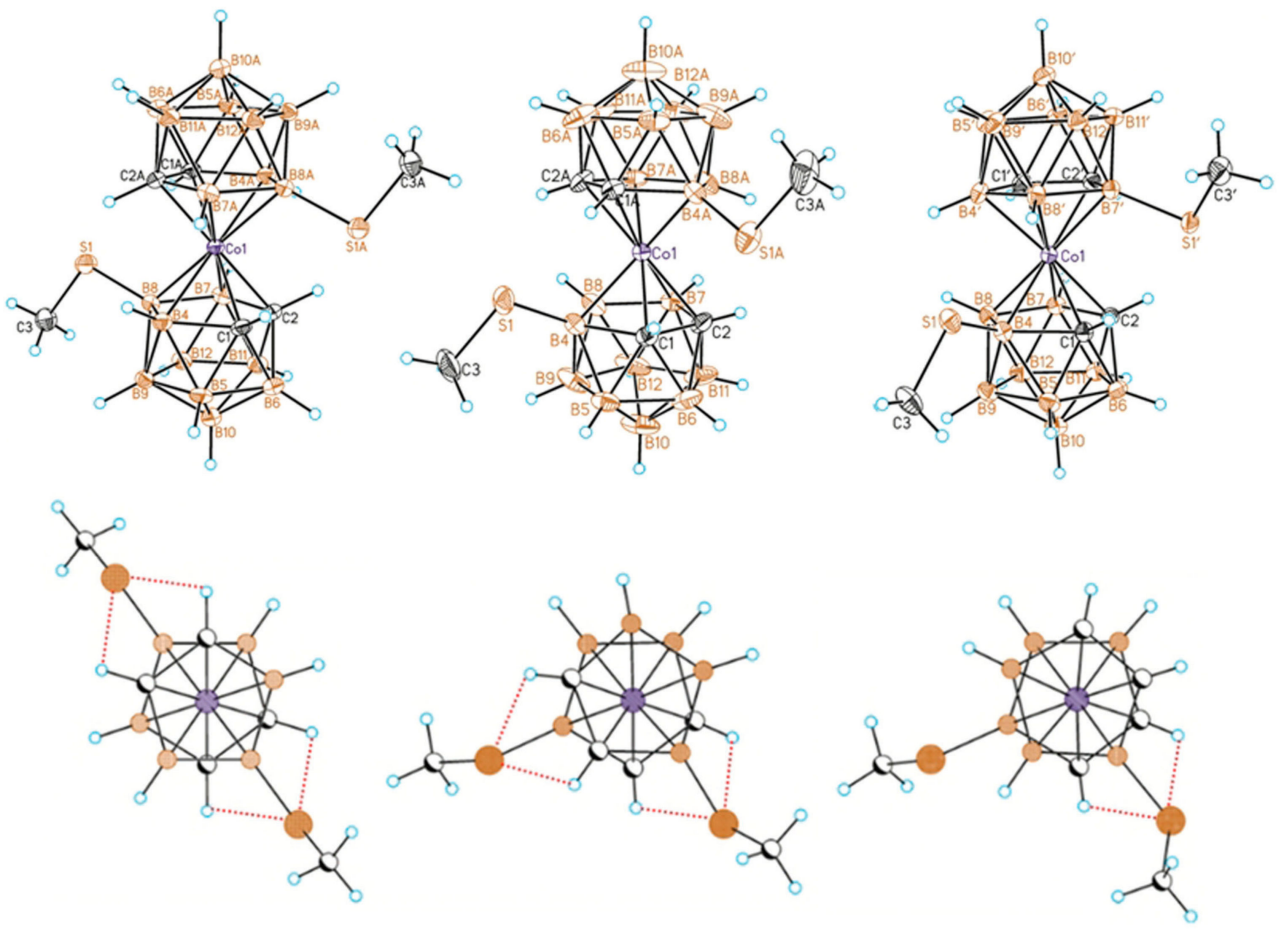
X-ray crystal structures (**top**) and intramolecular CH…S(Me)B hydrogen bonding (**bottom**) in methylsulfanyl derivatives of cobalt bis(dicarbollide) [8,8′-(MeS)_2_-3,3′-Co(1,2-C_2_B_9_H_10_)_2_]^−^, [4,4′-(MeS)_2_-3,3′-Co(1,2-C_2_B_9_H_10_)_2_]^−^ and [4,7′-(MeS)_2_-3,3′-Co(1,2-C_2_B_9_H_10_)_2_]^−^. Adopted with permission from [90]. Copyright (2017) John Wiley and Sons.

Recently, a series of the methylsulfanyl derivatives of cobalt bis(dicarbollide) containing the capable to coordination MeS groups at different positions of the upper belt of the dicarbollide ligands was synthesized starting from the corresponding *B*-methylslfanyl derivatives of *nido*-carborane [10-MeS-7,8-C_2_B_9_H_11_]^−^ [88] and [9-MeS-7,8-C_2_B_9_H_11_]^−^ [89]. The single crystal X-ray diffraction study of the *B*-methylsulfanyl derivatives revealed that the *transoid* and *gauche* conformations of the [8,8′-(MeS)_2_-3,3′-Co(1,2-C_2_B_9_H_10_)_2_]^−^ and [4,4′-(MeS)_2_-3,3′-Co(1,2-C_2_B_9_H_10_)_2_]^−^ isomers are stabilized by four intramolecular CH_carb_···S hydrogen bonds (2.683–2.712 and 2.709–2.752 Å, respectively), whereas the *gauche* conformation of the [4,7′-(MeS)_2_-3,3′-Co(1,2-C_2_B_9_H_10_)_2_]^−^ isomer is stabilized by two intramolecular CH_carb_…S hydrogen bonds (2.699–2.711 Å) (Figure 28) [90]. The NMR study suggests that the *transoid* conformation of the 8,8′-isomer is preserved in solution. These results agree well with the data of the DFT calculations [90].

**Scheme 11:**
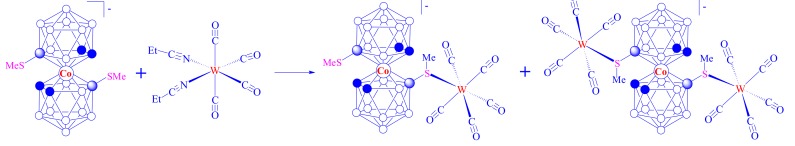
Synthesis of pentacarbonyl tungsten complexes of 8,8′-bis(methylsulfanyl) cobalt bis(dicarbollide).

The preliminary study demonstrates that the weak intramolecular CH_carb_…S(Me)B hydrogen bonds between the dicarbollide ligands can be disrupted in the complexation reaction with such rather weak electron acceptors as tungsten carbonyls (Scheme 11) [91].

Thus, with the proper choice of a coordinating metal fragment, 8,8′-bis(methylsulfanyl) cobalt bis(dicarbollide) can serve as a key structure fragment of the complexation-driven bistable molecular switches in which stabilization of the *transoid* conformation is due to intramolecular CH_carb_…S hydrogen bonding between the dicarbollide ligands, whereas its transformation to the *cisoid* conformation can be reached through formation of stronger dative S → M bonds on complexation with external metal complexes.

## 4. Conclusions

The design of rotatory molecular switches based on extremely stable sandwich organometallic complexes ferrocene and bis(dicarbollide) complexes of transition metals is reviewed. In the case of ferrocene-based molecular switches, stabilization of *syn*-conformers can be reached due to pH-driven intramolecular hydrogen bonding between substituents that capable to protonation or deprotonation or due to π-interactions between the (hetero)aromatic substutients in the cyclopentadienyl ligands. However, the stabilization of the *anti*-conformers is mostly based on repulsive interactions between the same-charged substituents, which does not always make it possible to clearly fix the angle of rotation of the ligands when repulsion weakens as the distance between the charges increases. This problem partially can be solved by introducing a second substituent into the cyclopentadienyl ring, which makes it possible to fix the angle of rotation of the ligands. Nevertheless, the internal drawback of such a design is the formation of a mixture of *rac*- and *meso*-diastereomers during synthesis of the corresponding 1,1′,3,3′-tetrasubstituted ferrocenes. This problem is absent in the case of molecular switches based on the 6,6′,8,8′-tetrasubstituted derivatives of transition metal bis(dicarbollide) complexes, where substituents at positions 8 and 8′ (“upper” belt) can be used for stabilization of *transoid*- or *cisoid*-conformers depending on the external conditions, whereas substituents at positions 6 and 6′ (“lower” belt) can serve for visualization of the rotation. The presented results demonstrate great potential of application of ferrocene and the iron group bis(dicarbollides) as rotary modules in the design of molecular switches. However, this area of research still remains in the shadow of the outstanding results obtained in the synthesis of purely organic molecular machines and therefore it needs a closer interest of the chemical community.

## References

[1] Feringa B.L. (2001). In control of motion: From molecular switches to molecular motors. Acc. Chem. Res..

[2] Sauvage J.-P. (2001). Molecular Machines and Motors (Structure and Bonding, Volume 99).

[3] Credi A., Silvi S., Venturi M. (2014). Molecular Machines and Motors: Recent Advances and Perspectives (Topics in Current Chemistry, Volume 354).

[4] Erbas-Cakmak S., Leigh D.A., McTernan C.T., Nussbaumer A.L. (2015). Artificial molecular machines. Chem. Rev..

[5] The Nobel Prize in Chemistry 2016. http://www.nobelprize.org/nobel_prizes/chemistry/laureates/2016/.

[6] Budyka M.F. (2017). Molecular switches and logic gates for information processing, the bottom-up strategy: From silicon to carbon, from molecules to supermolecules. Russ. Chem. Rev..

[7] Feringa B.L., Browne W.R. (2011). Molecular Switches.

[8] Canary J.W., Mortezaei S., Liang J. (2010). Transition metal-based chiroptical switches for nanoscale electronics and sensors. Coord. Chem. Rev..

[9] Werner H. (2012). At least 60 years of ferrocene: The discovery and rediscovery of the sandwich complexes. Angew. Chem. Int. Ed..

[10] Bohn R.K., Haaland A. (1966). On the molecular structure of ferrocene, Fe(C_5_H_5_)_2_. J. Organomet. Chem..

[11] Scottwell S.Ø., Crowley J.D. (2016). Ferrocene-containing non-interlocked molecular machines. Chem. Commun..

[12] Wang X.-B., Dai B., Woo H.-K., Wang L.-S. (2005). Intramolecular rotation through proton transfer: [Fe(η^5^-C_5_H_4_CO_2_^−^)_2_] versus [(η^5^-C_5_H_4_CO_2_^−^)Fe(η^5^-C_5_H_4_CO_2_H)]. Angew. Chem. Int. Ed..

[13] Palekik G.J. (1969). Crystal and molecular structure of ferrocenedicarboxylic acid. Inorg. Chem..

[14] Takusagawa F., Koetzle T.F. (1979). The crystal and molecular structure of 1,1′-ferrocene dicarboxylic acid (triclinic modification): Neutron and X-ray diffraction studies at 78 K and 298 K. Acta Cryst..

[15] Zakaria C.M., Ferguson G., Lough A.J., Glidewell C. (2002). Ferrocene-1,1′-dicarboxylic acid as a building block in supramolecular chemistry: Supramolecular structures in one, two and three dimensions. Acta Cryst..

[16] Horikoshi R., Mochida T. (2010). Ferrocene-containing coordination polymers: Ligand design and assembled structures. Eur. J. Inorg. Chem..

[17] Chandrasekhar V., Thirumoorthia R. (2010). Coordination polymers containing ferrocene backbone. Synthesis, structure and electrochemistry. Dalton Trans..

[18] Masello A., Sanakis Y., Boudalis A.K., Abboud K.A., Christou G. (2011). Iron(III) chemistry with ferrocene-1,1′-dicarboxylic acid (fdcH_2_): An Fe_7_ cluster with an oxidized fdc^−^ ligand. Inorg. Chem..

[19] Hirai K., Uehara H., Kitagawa S., Furukawa S. (2012). Redox reaction in two-dimensional porous coordination polymers based on ferrocenedicarboxylates. Dalton Trans..

[20] Masello A., Abboud K.A., Wernsdorfer W., Christou G. (2013). Mn_8_ cluster with ferrocene-1,1′-dicarboxylate ligation: Single-molecule magnetism with multiple external redox centers. Inorg. Chem..

[21] Chandrasekhar V., Chakraborty A., Sañudoc E.C. (2013). Ferrocene-based compartmental ligand for the assembly of neutral Zn^II^/Ln^III^ heterometallic complexes. Dalton Trans..

[22] Chakraborty A., Bag P., Rivière E., Mallah T., Chandrasekhar V. (2014). Assembly of heterobimetallic Ni^II^–Ln^III^ (Ln^III^ = Dy^III^, Tb^III^, Gd^III^, Ho^III^, Er^III^, Y^III^) complexes using a ferrocene ligand: Slow relaxation of the magnetization in Dy^III^, Tb^III^ and Ho^III^ analogues. Dalton Trans..

[23] Ospina Castro M.L., Briceño A., González T., Reiber A., Jorge G., Duran M.H. (2015). Metallic macrocycles built-up from ferrocenedicarboxylate and ancillary nitrogen heterocycles: Hydrothermal synthesis, crystal structures and electrochemical properties. Inorg. Chim. Acta.

[24] Ospina-Castro M.L., Reiber A., Jorge G., Ávila E.E., Briceño A. (2017). Novel 3-D interpenetrated metal-organometallic networks based on self-assembled Zn(II)/Cu(II) from 1,1′-ferrocenedicarboxylic acid and 4,4′-bipyridine. CrystEngComm.

[25] Yanilkin V.V., Nasybullina G.R., Nastapova N.V., Madzhidov T.I., Latypova L.Z., Morozov V.I., Shekurov R.P., Milyukov V.A. (2015). Electrochemically driven molecular rotors based on ferrocene-1,1′-diyl-bisphosphinic acids. Russ. J. Electrochem..

[26] Shekurov R.P., Milyukov V.A., Islamov D.P., Krivolapov D.B., Kataeva O.N., Gerasimova T.P., Katsyuba S.A., Nasybullina G.R., Yanilkin V.V., Sinyashin O.G. (2014). Synthesis and structure of ferrocenylphosphonic acids. J. Organomet. Chem..

[27] Shekurov R.P., Tufatullin A.I., Milyukov V.A., Kataeva O.N., Sinyashin O.G. (2014). Supramolecular architecture of diammonium ferrocene-1,1′-diyldiphosphinates. Russ. Chem. Bull..

[28] Toyama T., Komori S., Yoshino J., Hayashi N., Higuchi H. (2013). Synthesis and properties of 1,1′-bis[*p*-(*N*,*N*-dimethylaminophenyl)butadiynyl]ferrocene: A methodology for proton-mediated reversible conformation control of two function sites. Tetrahedron Lett..

[29] Moriuchi T., Nomoto A., Yoshida K., Ogawa A., Hirao T. (2001). Chirality Organization of ferrocenes bearing podand dipeptide chains: Synthesis and structural characterization. J. Am. Chem. Soc..

[30] Moriuchi T., Hirao T. (2010). Design of ferrocene-dipeptide bioorganometallic conjugates to induce chirality-organized structures. Acc. Chem. Res..

[31] Moriuchi T., Nishiyama T., Nobu M., Hirao T. (2017). Control of helical chirality of ferrocene-dipeptide conjugates by the secondary structure of dipeptide chains. Chem. Eur. J..

[32] Deck P.A., Lane M.J., Montgomery J.L., Slebodnick C., Fronczek F.R. (2000). Synthesis and structural trends in pentafluorophenyl-substituted ferrocenes, 1,4-tetrafluorophenylene-linked diferrocenes, and 1,1′-ferrocenylene-1,4-tetrafluorophenylene co-oligomers. Organometallics.

[33] Baumgardt I., Butenschön H. (2010). 1,1′-Diaryl-substituted ferrocenes: Up to three hinges in oligophenylene-ethynylene-type molecular wires. Eur. J. Org. Chem..

[34] Enders M., Kohl G., Pritzkow H. (2001). Synthesis and coordination behaviour of the new (8-quinolyl)-cyclopentadienyl ligand. J. Organomet. Chem..

[35] Crowley J.D., Steele I.M., Bosnich B. (2006). Protonmotive force: Development of electrostatic drivers for synthetic molecular motors. Chem. Eur. J..

[36] Siemeling U., Vorfeld U., Neumann B., Stammler H.-G., Fontani M., Zanello P. (2001). Synthesis and crystal structure of 1,1′-bis[4-(2,2′:6′,2″-terpyridin-4′-yl)phenyl]-octamethylferrocene. J. Organomet. Chem..

[37] Scottwell S.Ø., Elliott A.B.S., Shaffer K.J., Nafady A., McAdam C.J., Gordon K.C., Crowley J.D. (2015). Chemically and electrochemically induced expansion and contraction of a ferrocene rotor. Chem. Commun..

[38] Scottwell S.Ø., Barnsley J.E., McAdam C.J., Gordon K.C., Crowley J.D. (2017). A ferrocene based switchable molecular folding ruler. Chem. Commun..

[39] Zhang D., Zhang Q., Su J., Tian H. (2009). A dual-ion-switched molecular brake based on ferrocene. Chem. Commun..

[40] Iordache A., Oltean M., Milet A., Thomas F., Baptiste B., Sain-Aman E., Bucher C. (2012). Redox control of rotary motions in ferrocene-based elemental ball bearing. J. Am. Chem. Soc..

[41] Wang W.-Y., Ma N.-N., Sun S.L., Qiu Y.-Q. (2014). Redox control of ferrocene-based complexes with systematically extended π-conjugated connectors: Switchable and tailorable second order nonlinear optics. Phys. Chem. Chem. Phys..

[42] Takai A., Yasuda T., Ishizuka T., Kojima T., Takeuchi M. (2013). A directly linked ferrocene-naphthalenediimide conjugate: Precise control of stacking structures of π-systems by redox stimuli. Angew. Chem. Int. Ed..

[43] Takai A., Kajitani T., Fukushima T., Kishikawa K., Yasuda T., Takeuchi M. (2016). Supramolecular assemblies of ferrocene-hinged naphthalenediimides: Multiple conformational changes in film states. J. Am. Chem. Soc..

[44] Kinbara K., Muraoka T., Aida T. (2008). Chiral ferrocenes as novel rotary modules for molecular machines. Org. Biomol. Chem..

[45] Muraoka T., Kinbara K., Kobayashi Y., Aida T. (2003). Light-driven open-close motion of chiral molecular scissors. J. Am. Chem. Soc..

[46] Muraoka T., Kinbara K., Wakamiya A., Yamaguchi S., Aida T. (2007). Crystallographic and chiroptical studies on tetraarylferrocenes for use as chiral rotary modules for molecular machines. Chem. Eur. J..

[47] Muraoka T., Kinbara K., Aida T. (2007). Reversible operation of chiral molecular scissors by redox and UV light. Chem. Commun..

[48] Muraoka T., Kinbara K., Aida T. (2006). Mechanical twisting of a guest by a photoresponsive host. Nature.

[49] Muraoka T., Kinbara K., Aida T. (2006). A self-locking molecule operative with a photoresponsive key. J. Am. Chem. Soc..

[50] Kai H., Nara S., Kinbara K., Aida T. (2008). Toward long-distance mechanical communication: Studies on a ternary complex interconnected by a bridging rotary module. J. Am. Chem. Soc..

[51] Tanaka K., Kinbara K. (2008). Toward autonomously operating molecular machines driven by transition-metal catalyst. Mol. BioSyst..

[52] Allinger N.L., Eliel E.L., Schlögl K., Allinger N.L., Eliel E.L. (1967). Stereochemistry of Metallocenes. Topics in Stereochemistry, Volume 1.

[53] Li H., Zhang H., Zhang Q.-W., Qu D.-H. (2012). A switchable ferrocene-based [1]rotaxane with an electrochemical signal output. Org. Lett..

[54] Hawthorne M.F., Young D.C., Wegner P.A. (1965). Carbametallic boron hydride derivatives. I. Apparent analogs of ferrocene and ferricinium ion. J. Am. Chem. Soc..

[55] Hawthorne M.F., Andrews T.D. (1965). Carborane analogues of cobalticinium ion. Chem. Commun..

[56] Sivaev I.B., Bregadze V.I. (1999). Chemistry of cobalt bis(dicarbollides). A review. Collect. Czech. Chem. Commun..

[57] Sivaev I.B., Bregadze V.I. (2000). Chemistry of nickel and iron bis(dicarbollides). A review. J. Organomet. Chem..

[58] Dash B.P., Satapathy R., Swain B.R., Mahanta C.S., Jena B.B., Hosmane N.S. (2017). Cobalt bis(dicarbollide) anion and its derivatives. J. Organomet. Chem..

[59] Bühl M., Hnyk D., Macháček J. (2005). Computational study of structures and properties of metallaboranes: Cobalt bis(dicarbollide). Chem. Eur. J..

[60] Bühl M., Holub J., Hnyk D., Macháček J. (2006). Computational studies of structures and properties of metallaboranes. 2. Transition-metal dicarbollide complexes. Organometallics.

[61] Hawthorne M.F., Zink J.I., Skelton J.M., Bayer M.B., Liu C., Livshits E., Baer R., Neuhauser D. (2004). Electrical or photocontrol of the rotary motion of a metallacarborane. Science.

[62] Warren L.F., Hawthorne M.F. (1970). Chemistry of the bis[π-(3)-1,2-dicarbollyl] metalates of nickel and palladium. J. Am. Chem. Soc..

[63] Hawthorne M.F., Ramachandran B.M., Kennedy R.D., Knobler C.B. (2006). Approaches to rotary molecular motors. Pure Appl. Chem..

[64] Safronov A.V., Shlyakhtina N.I., Everett T.A., VanGordon M.R., Sevryugina Y.V., Jalisatgi S.S., Hawthorne M.F. (2014). Direct observation of bis(dicarbollyl)nickel conformers in solution by fluorescence spectroscopy: An approach to redox-controlled metallacarborane molecular motors. Inorg. Chem..

[65] Shlyakhtina N.I., Safronov A.V., Sevryugina Y.V., Jalisatgi S.S., Hawthorne M.F. (2015). Synthesis, characterization, and preliminary fluorescence study of a mixed ligand bis(dicarbollyl)nickel complex bearing a tryptophan-BODIPY FRET couple. J. Organomet. Chem..

[66] Kirillova N.I., Zhdanov A.S., Gusev A.I., Kirin V.N., Knyazev S.P., Sokolova T.V. (1989). The molecular structures of the dicarbaboryl derivatives [3,3′-M(8,1,2-ClC_2_B_9_H_10_)_2_]K. Organomet. Chem. USSR.

[67] Hurlburt P.K., Miller R.L., Abney K.D., Foreman T.M., Butcher R.J., Kinkead S.A. (1995). New synthetic routes to B-halogenated derivatives of cobalt dicarbollide. Inorg. Chem..

[68] Kazheva O.N., Kravchenko A.V., Aleksandrov G.G., Sivaev I.B., Bregadze V.I., Kosenko I.D., Lobanova I.A., Buravov L.I., Starodub V.A., Dyachenko O.A. (2014). Syntheses, structures, and electroconductivity of bis(ethylenedithio)tetrathiafulvalene (BEDT-TTF) and bis(methylenedithio)tetrathiafulvalene (BMDT-TTF) salts with cobalt 8,8′-dichloro-3,3′-bis(1,2-dicarbollide). Russ. Chem. Bull..

[69] Kazheva O., Alexandrov G., Kravchenko A., Starodub V., Lobanova I., Sivaev I., Bregadze V., Buravov L., Dyachenko O. (2008). First molecular conductors with 8,8′-dibromo cobalt bis(dicarbollide) anion. Solid State Sci..

[70] Sivy P., Preisinger A., Baumgartner O., Valach F., Koreñ B., Matel L. (1986). Structure of caesium 3,3′-*commo*-bis(decahydro-8-iodo-1,2-dicarba-3-cobalta-*closo*-dodecaborate)(1-). Acta Cryst. C.

[71] Kazheva O.N., Alexandrov G.G., Kravchenko A.V., Starodub V.A., Lobanova I.A., Sivaev I.B., Bregadze V.I., Titov L.V., Buravov L.I., Dyachenko O.A. (2009). Molecular conductors with 8,8’-diiodo cobalt bis(dicarbollide) anion. J. Organomet. Chem..

[72] Kazheva O.N., Kravchenko A.V., Aleksandrov G.G., Kosenko I.D., Lobanova I.A., Bregadze V.I., Chudak D.M., Buravov L.I., Protasova S.G., Starodub V.A., Dyachenko O.A. (2016). Synthesis, structure, and properties of a new bifunctional radical cation salt with ferracarborane anion: (BEDT-TTF)_2_[8,8′-Cl_2–3_,3′-Fe(1,2-C_2_B_9_H_10_)_2_]. Russ. Chem. Bull..

[73] Kazheva O.N., Kravchenko A.V., Kosenko I.D., Alexandrov G.G., Chudak D.M., Starodub V.A., Lobanova I.A., Bregadze V.I., Buravov L.I., Protasova S.G., Dyachenko O.A. (2017). First hybrid radical-cation salts with halogen substituted iron bis(dicarbollide) anions—Synthesis, structure, properties. J. Organomet. Chem..

[74] Pauling L. (1960). The Nature of the Chemical Bond.

[75] Gashti A.N., Huffman J.C., Edwards A., Szekeley G., Siedle A.R., Karty J.A., Reilly J.P., Todd L.J. (2000). Fluorination studies of the [*commo*-3,3′-Co(3,1,2-CoC_2_B_9_H_11_)_2_]^−^ ion. J. Organomet. Chem..

[76] Kazheva O.N., Alexandrov G.G., Kravchenko A.V., Sivaev I.B., Starodub V.A., Kosenko I.D., Lobanova I.A., Bregadze V.I., Buravov L.V., Dyachenko O.A. (2015). New organic conductors with halogen and phenyl substituted cobalt bis(dicarbollide) anions. J. Chem. Eng. Chem. Res..

[77] Sivy P., Preisinger A., Baumgartner O., Valach F., Koreñ B., Matel L. (1986). Structure of caesium 8-iodo-3,3′-*commo*-bis(decahydro-1,2-dicarba-3-cobalta-*closo*-dodecaborate)(1-). Acta Cryst. C.

[78] Kazheva O.N., Alexandrov G.G., Kravchenko A.V., Starodub V.A., Sivaev I.B., Lobanova I.A., Bregadze V.I., Buravov L.I., Dyachenko O.A. (2007). New fulvalenium salts of bis(dicarbollide) cobalt and iron: Synthesis, crystal structure and electrical conductivity. J. Organomet. Chem..

[79] Kazheva O.N., Alexandrov G.G., Kravchenko A.V., Kosenko I.D., Lobanova I.A., Sivaev I.B., Filippov O.A., Shubina E.S., Bregadze V.I., Starodub V.A. (2011). Molecular conductors with a 8-hydroxy cobalt bis(dicarbollide) anion. Inorg. Chem..

[80] Rokitskaya T.I., Kosenko I.D., Sivaev I.B., Antonenko Y.N., Bregadze V.I. (2017). Fast flip-flop of halogenated cobalt bis(dicarbollide) anion in a lipid bilayer membrane. Phys. Chem. Chem. Phys..

[81] Plešek J., Heřmánek S., Franken A., Císařová I., Nachtigal C. (1997). Dimethyl sulfate induced nucleophilic substitution of the [bis(1,2-dicarbollido)-3-cobalt(1-)]ate ion. Syntheses, properties and structures of its 8,8′-μ-sulfato,8-phenyl and 8-dioxane derivatives. Collect. Czech. Chem. Commun..

[82] Rojo I., Teixidor F., Viñas C., Kivekäs R., Sillanpää R. (2003). Relevance of the electronegativity of boron in η^5^-coordinating ligands: Regioselective monoalkylation and monoarylation in cobaltabisdicarbollide [3,3′-Co(1,2-C_2_B_9_H_11_)_2_]^−^clusters. Chem. Eur. J..

[83] Bregadze V.I., Kosenko I.D., Lobanova I.A., Starikova Z.A., Godovikov I.A., Sivaev I.B. (2010). C-H Bond activation of arenes by [8,8′-μ-I-3,3′-Co(1,2-C_2_B_9_H_10_)_2_] in the presence of sterically hindered Lewis bases. Organometallics.

[84] Kosenko I.D., Lobanova I.A., Godovikov I.A., Starikova Z.A., Sivaev I.B., Bregadze V.I. (2012). Mild C-H activation of activated aromatics with [8,8′-μ-I-3,3′-Co(1,2-C_2_B_9_H_10_)_2_]: Just mix them. J. Organomet. Chem..

[85] Rojo I., Teixidor F., Viñas C., Kivekäs R., Sillanpää R. (2004). Synthesis and coordinating ability of an anionic cobaltabisdicarbollide ligand geometrically analogous to BINAP. Chem. Eur. J..

[86] Teixidor F., Pedrajas J., Rojo I., Viñas C., Kivekäs R., Sillanpää R., Sivaev I., Bregadze V., Sjöberg S. (2003). Chameleonic capacity of [3,3’-Co(1,2-C_2_B_9_H_11_)_2_]^−^ in coordination. Generation of the highly uncommon S(thioether)-Na bond. Organometallics.

[87] Kazakov G.S., Stogniy M. Y., Sivaev I.B., Suponitsky K.Y., Godovikov I.A., Kirilin A.D., Bregadze V.I. (2015). Synthesis of crown ethers with the incorporated cobalt bis(dicarbollide) fragment. J. Organomet. Chem..

[88] Anufriev S.A., Sivaev I.B., Suponitsky K.Y., Godovikov I.A., Bregadze V.I. (2017). Synthesis of 10-methylsulfide and 10-alkylmethylsulfonium *nido*-carborane derivatives: B-H…π interactions between the B-H-B hydrogen atom and alkyne group in 10-RC≡CCH_2_S(Me)-7,8-C_2_B_9_H_11_. Eur. J. Inorg. Chem..

[89] Anufriev S.A., Sivaev I.B., Suponitsky K.Y., Bregadze V.I. (2017). Practical synthesis of 9-methylthio-7,8-*nido*-carborane [9-MeS-7,8-C_2_B_9_H_11_]^−^. Some evidences of BH … X hydride-halogen bonds in 9-XCH_2_(Me)S-7,8-C_2_B_9_H_11_ (X = Cl, Br, I). J. Organomet. Chem..

[90] Anufriev S.A., Erokhina S.A., Suponitsky K.Y., Godovikov I.A., Filippov O.A., Fabrizi de Biani F., Corsini M., Chizhov A.O., Sivaev I.B. (2017). Methylsulfanyl-stabilized rotamers of cobalt bis(dicarbollide). Eur. J. Inorg. Chem..

[91] Timofeev S.V., Anufriev S.A., Sivaev I.B., Bregadze V.I. Synthesis of pentacarbonyl tungsten complexes of 8,8′-dimethylsulfanyl cobalt bis(dicarbollide). Russ. Chem. Bull..

